# A randomized, double-blind, placebo-controlled, dose-escalating phase I trial to evaluate safety and immunogenicity of a plant-produced, bivalent, recombinant norovirus-like particle vaccine

**DOI:** 10.3389/fimmu.2022.1021500

**Published:** 2022-10-07

**Authors:** Isabel Leroux-Roels, Cathy Maes, Jasper Joye, Bart Jacobs, Franziska Jarczowski, André Diessner, Yorick Janssens, Gwenn Waerlop, Kirsi Tamminen, Suvi Heinimäki, Vesna Blazevic, Geert Leroux-Roels, Victor Klimyuk, Hiroshi Adachi, Kazuyuki Hiruta, Frank Thieme

**Affiliations:** ^1^ Center for Vaccinology (CEVAC), Ghent University and University Hospital, Ghent, Belgium; ^2^ Icon Genetics GmbH, a Denka Company, Halle, Germany; ^3^ Vaccine Research Center, University of Tampere, Tampere, Finland; ^4^ Denka Co., Ltd., Tokyo, Japan

**Keywords:** norovirus, virus-like particle, plant-produced, vaccine, clinical trial, adults, safety, immunogenicity

## Abstract

Noroviruses (NoV) are the leading cause of epidemic acute gastroenteritis in humans worldwide and a safe and effective vaccine is needed. Here, a phase I, double-blind, placebo-controlled clinical trial was performed in 60 healthy adults, 18 to 40 years old. Safety (primary objective) and immunogenicity (secondary and exploratory objectives) of a bivalent (GI.4 and GII.4), plant-produced, virus-like particle (VLP), NoV vaccine candidate formulation were investigated at two dose levels (50 µg + 50 µg and 150 µg + 150 µg) without adjuvant. Overall, 13 subjects (65.0%) in the 50 µg group, 16 subjects (80.0%) in the 150 µg group, and 14 subjects (70.0%) in the placebo group reported at least 1 solicited local or general symptom during the 7-day post-vaccination periods following each dose. Severe solicited adverse events (AEs) were rare (2 events in the 50 µg group). A total of 8 subjects (40.0%) in each group reported at least one unsolicited AE during the 28-day post-vaccination periods. Immunogenicity was assessed on days 1, 8, 29, 57, 183 and 365. All subjects were pre-exposed to norovirus as indicated by baseline levels of the different immunological parameters examined. Vaccine-specific humoral and cellular immune responses increased after the first dose but did not rise further after the second vaccination. Increased GI.4- and GII.4-specific IgG titers persisted until day 365. The vaccine elicited cross-reactive IgG antibodies against non-vaccine NoV VLPs, which was more pronounced for NoV strains of the same genotype as the GII.4 vaccine strain than for non-vaccine genotypes. Significant blocking anti-GI.4 and anti-GII.4 VLP titers were triggered in both dose groups. Lymphoproliferation assays revealed strong cell-mediated immune responses that persisted until day 365. In conclusion, both dose levels were safe and well-tolerated, and no higher incidence of AEs was observed in the higher dose group. The data show that a single dose of the vaccine formulated at 50 µg of each VLP is sufficient to reach a peak immune response after 8 to 28 days. The results of this Phase I study warrant further evaluation of the non-adjuvanted vaccine candidate.

**Clinical trial registration:**

https://clinicaltrials.gov/ct2/show/record/NCT05508178, identifier (NCT05508178).

## Introduction

Noroviruses (NoV) are the leading cause of sporadic and epidemic acute non-bacterial gastroenteritis and foodborne diarrhoeal diseases in humans worldwide. NoV is associated with nearly 20% of diarrheal diseases globally (1, 2). Noroviruses are estimated to cause a median of 669 million illnesses and 219,000 deaths across all age groups per year globally (1) resulting in a total of 4.2 billion US dollar in direct health system costs (outpatient visits and hospitalization) and 60.3 billion US dollar in societal costs (productivity losses due to absenteeism or mortality) per year (3). Severe outbreaks typically occur in close-quartered environments such as hospitals, military barracks, schools, camps, and ships (4–7). Infection is characterized by severe vomiting, diarrhea, and abdominal cramping for 28 – 60 hours within 10 – 51 hours of exposure (8). The virus is transmitted by the fecal/oral route and virus particles exhibit high environmental stability on exposed surfaces (9). Hygiene measures and social distancing during the Covid-19 pandemic could greatly reduce outbreaks in the years 2020 and 2021. However, since restrictions have been lifted in 2022, fast resurgence of norovirus has been observed as predicted (10, 11).

Noroviruses are a group of non-enveloped, positive-sense, single-stranded, ribonucleic acid (RNA) viruses. The RNA genome consists of three open reading frames (ORFs) of which ORF2 encodes the major capsid protein VP1 that determines the antigenicity of the virus (12). Noroviruses are classified based on phylogenetic clustering of the complete VP1 amino acid sequence into 10 genogroups (GI to GX) and 49 confirmed genotypes (13). GI and GII genogroups have been responsible for most human diseases (12). In the past two decades GII.4 viruses have caused the majority of norovirus outbreaks (12).

No approved vaccines against norovirus are currently available. Considering the burden of disease caused by norovirus, the development of an efficacious vaccine to prevent severe norovirus gastroenteritis is of high priority. Several candidate vaccines are under development, and projections indicate that an efficacious vaccine could have both clinical and economic benefits (14). However, development of such a vaccine is hampered by factors like the incomplete understanding of protective immunity to norovirus and the lack of a permissive cell culture system and suitable animal models (15). The most advanced candidate vaccines are based on virus-like particles (VLPs) formed by norovirus capsid protein VP1 formulated with alum adjuvant. Clinical trials in adult volunteers (16–19) and children (20) have demonstrated their safety and immunogenicity.

Pre-clinical studies of the vaccine candidate evaluated here showed safety and induction of strong genogroup-specific immune responses by vaccination without adjuvant (21). Furthermore, clinical studies by Takeda/HilleVax with norovirus VLP-based vaccines in a previously exposed population showed little effect of the tested adjuvants (17, 22). Consequently, the vaccine candidate was formulated without adjuvant.

This clinical trial investigates the first plant-produced, prophylactic vaccine candidate manufactured in Europe. Worldwide more and more vaccine candidates are manufactured in plants due to the advantageous safety profile, flexibility, speed, scalability and economics of transient, green plant-based expression systems (23–28). The goals of this trial were to evaluate the safety and immunogenicity of a non-adjuvanted, bivalent [GI.4 Chiba 407 (1987) + GII.4 Aomori 2 (2006)] vaccine consisting of VLPs at two dose levels (50 µg or 150 µg each) manufactured using the magnICON^®^ plant-based, transient expression system (29, 30). The current Good Manufacturing Practice (GMP) compliant magnICON system has been used to manufacture vaccines and therapeutic antibodies that have demonstrated safety in prior clinical studies (31, 32).

## Materials and methods

### Study vaccine

**Figure 1:**
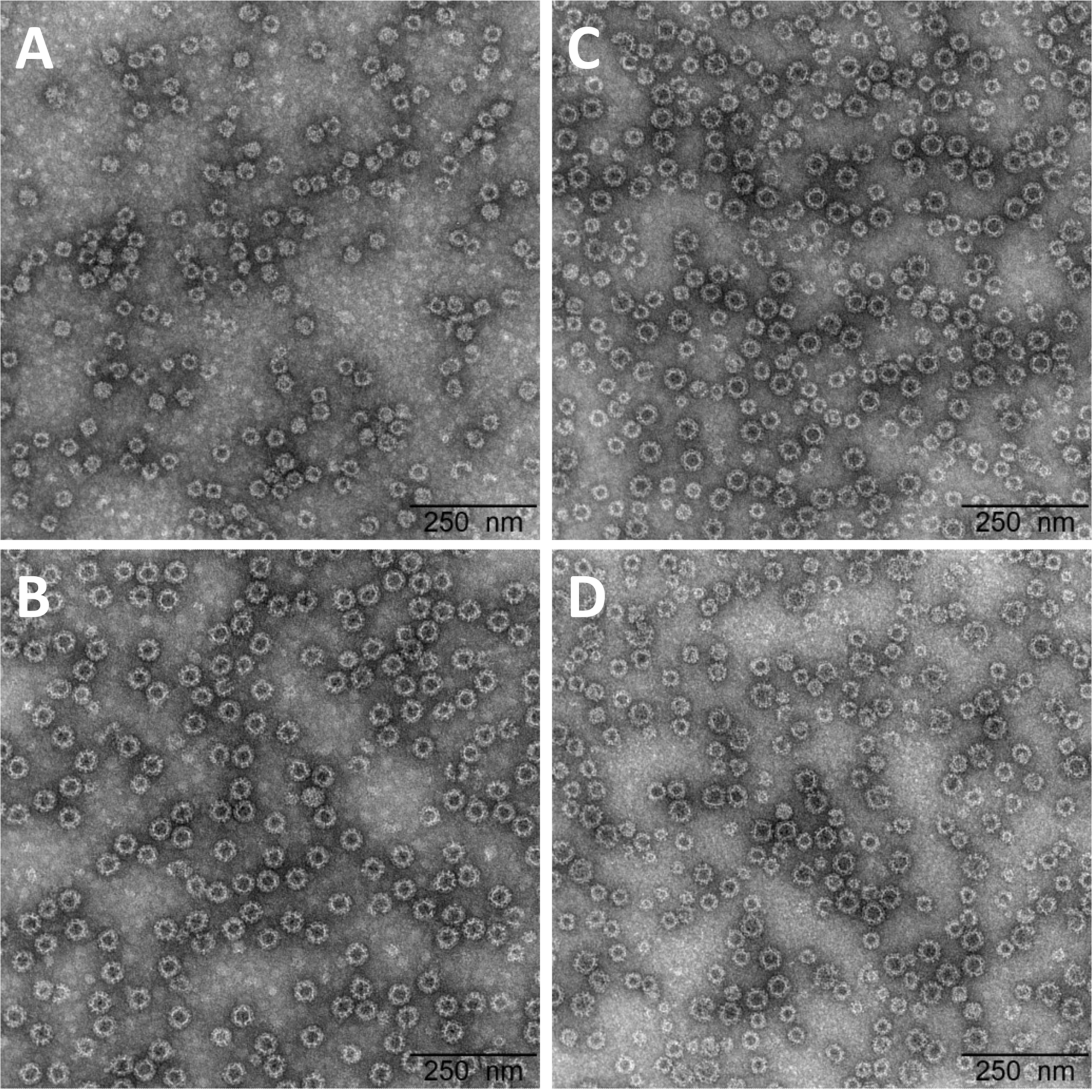
Electron micrographs of the highly purified, plant-produced, norovirus-like particles and vaccine formulations. VLPs formed by **(A)** GI.4 Chiba 407 (1987) capsid protein VP1 (batch IN003); **(B)** GII.4 Aomori 2 (2006) capsid protein VP1 (batch IN002); and formulated vaccine (1:1 mixture of both) at **(C)** 50 µg + 50 µg (batch IN004) and **(D)** 150 µg + 150 µg (batch IN005). Clinical batches were examined by transmission electron microscopy following negative staining with uranyless. The black bar corresponds to 250 nm. VLP, virus-like particle.

The recombinant norovirus vaccine candidate, rNV-2v, consists of VLPs self-assembled *in vitro* from the major capsid proteins (VP1) of NoV strain GI.4 Chiba 407 (1987) and strain GII.4 Aomori 2 (2006), respectively. The VLPs resemble the structure of the intact NoV capsids (**Figure 1**). The VP1 antigens were recombinantly manufactured by Icon Genetics GmbH using its proprietary magnICON^®^ technology in green plants (21). rNV-2v was produced in compliance with GMP guidelines and provided as pre-mixed vaccine in sterile buffered aqueous solution for intramuscular injection. The vaccine vehicle was used as placebo. The following rNV-2v and placebo batches were used in this trial: IN004 NV003002 (rNV-2v: 50 µg GI.4 + 50 µg GII.4); IN005 NV003002 (rNV-2v: 150 µg GI.4 + 150 µg GII.4); IN006 (Placebo).

### Study design and participants

This randomized, placebo-controlled, double-blind, dose-escalating Phase I study was performed at one single center (Center for Vaccinology, Ghent University Hospital, Ghent, Belgium) between 17 August 2020 and 26 October 2021 (26 August 2020 First Subject First Dose and 26 November 2020 Last Subject Last Dose) in accordance with Good Clinical Practice. The study was approved by the Ethics Committee of the Ghent University Hospital and the Belgian Federal Agency for Medicines and Health Products (FAMHP) (EudraCT number: 2019-003226-25). Written informed consent was obtained from all participants.

Sixty (60) healthy men and women aged ≥18 to ≤40 years were enrolled in the study. Eligibility was based on the outcome of a screening visit that preceded the day of first vaccine administration by 28 to 1 days. Health status was based on medical anamnesis, physical examination and a laboratory test panel. Standard in- and exclusion criteria have been applied (**Supplementary Table 1**). The participants enrolled in the study were randomly assigned to one of two sequential study cohorts. Cohort 1 consisted of 30 participants of whom 10 received a placebo solution and 20 received low dose vaccine (50 µg VLP GI.4 and 50 µg VLP GII.4). Cohort 2 consisted of 30 participants of whom 10 received a placebo solution and 20 received high dose vaccine (150 µg VLP GI.4 and 150 µg VLP GII.4). Vaccines (or placebo) were administered in the deltoid muscle of the non-dominant arm at 0.5 mL per injection with 28 days ( ± 3 days) interval between dose 1 and dose 2. The subject and those responsible for the evaluation of any study endpoint were all unaware whether vaccine or placebo was administered. To do so, vaccine preparation and administration was done by authorized medical personnel who did not participate in any of the study clinical evaluations.

As per EMA guideline on “first in human” (FIH) studies, sentinel dosing was utilized in both cohorts. The first 3 participants (2 active and 1 placebo) in each cohort were dosed on day 1. Following a review of the safety data obtained after at least 48 hours by the Investigator, and in the absence of any safety concerns, 3 additional participants (2 active and 1 placebo) were dosed. The safety data of all sentinel participants were obtained through a safety phone call at least 48 hours after dosing. All safety data of the first 6 participants were reviewed by the Investigator. If no safety or tolerability concerns arose during this review, the remainder of the participants in the cohort (24 participants) were dosed. No more than 12 participants were dosed on a single day. The same sentinel strategy was applied for the second dose in both cohorts. An independent Safety Review Committee (SRC) reviewed all available safety data of at least 20 of the 30 participants in Cohort 1 and Cohort 2 from the first 7 days post first dose of the vaccine, before proceeding to the second dose. The SRC also reviewed available safety data from the 30 participants in Cohort 1 (all data up to 7 days post second dose of the vaccine), before proceeding to Cohort 2. For schematic representation of the study design see **Supplementary Figures 1, 2**.

In addition to the screening visit (day -28 to day -1) the study involved 7 more visits: Day 1 (1^st^ vaccine administration), day 8, day 29 (2^nd^ vaccine administration), day 36, day 57, day 183 and day 365 (end of study visit). The total study duration for each participant ranged between 14 and 15 months. For detailed schedule of assessment see **Supplementary Table 2**.

### Monitoring of safety and tolerability

A diary card was used to evaluate the occurrence of solicited adverse events (AEs) within 7 days after 1^st^ and 2^nd^ vaccine administration. These encompassed solicited local symptoms (pain, redness, and swelling at the injection site) and systemic signs and symptoms (fever [oral temperature], fatigue, gastrointestinal symptoms [nausea, vomiting, diarrhoea, and/or abdominal pain], headache, myalgia, shivering, and arthralgia). Incidence of abnormal haematology and serum chemistry laboratory values was examined in blood samples collected on day 1, 8, 29, 36, and 57. Unsolicited AEs were recorded through open-ended inquiries for 28 days after each vaccine administration. Intensities of AEs were monitored throughout the active phase and graded as mild, moderate, severe, or potentially life-threatening according to FDA guidance (33). Serious AEs (SAEs) were monitored throughout the study to month 12 (end of study visit).

### Monitoring of immunogenicity

Humoral immune responses were measured using serum samples prepared from venous blood ( ± 12 mL) drawn on day 1 (before the 1^st^ vaccine administration), day 8, day 29 (before the 2^nd^ vaccine administration) and subsequently on days 57, 183 and 365. Serum was prepared and kept frozen at –20 °C until analysed by ELISA.

Cellular immune responses were measured using peripheral blood mononuclear cells (PBMC) isolated from heparinized venous blood samples (± 50mL) collected on days 1, 29, 57 and 365. PBMC were isolated by isopycnic density centrifugation (Ficoll-Hypaque) and stored at –196 °C in liquid nitrogen until analysed by lymphoproliferation assay.

### IgG antibodies against vaccine VLPs GI.4 Chiba 407 (1987) and GII.4 Aomori 2 (2006)

Plant-produced VLPs of GI.4 Chiba 407 (1987) and GII.4 Aomori 2 (2006) (Icon Genetics GmbH) were coated onto separate 96-polystyrene plates (Nunc MaxiSorp) by adding 100 µL per well of a coating solution (4 µg/mL). Standard, samples and controls were added in eight serial two-fold dilutions to the plate (100 µL per well) and incubated for 2 to 2.5 hours at +37°C. Plates were washed 3 times with wash buffer before the polyclonal rabbit anti-human IgG detection antibody conjugated to HRP (horseradish peroxidase, Agilent) was added (100 µL/well). Plates were incubated for 60 to 75 minutes at room temperature on an orbital shaker and washed again 4 times with wash buffer. Next, 100 µL of the chromogen substrate TMB (Sigma) was added per well and incubated for 15 minutes at room temperature (RT) in the dark. The colorimetric reaction was stopped by adding 50 µL/well of the stopping solution consisting of 1N sulphuric acid (VWR). Read-out was performed within 30 minutes with a microtiter plate reader at 450 nm (Versamax, Molecular Devices). Optical Densitiy (OD) values were processed with a data reduction software (SoftMax Pro, Molecular Devices) using a 4-parameter logistic fitting algorithm to the standard curve, allowing the determination of antibody concentration in the samples, expressed as EU/mL.

As no international standard is available for the determination of norovirus-specific antibodies in serum, a pool of human serum samples (CEVAC) was used to constitute the reference standard for both enzyme-linked immunosorbent assays (ELISAs). This standard was attributed a concentration of 7 and 18 ELISA units per mL (EU/mL) for the GI.4 and GII.4-specific ELISA, respectively. Negative control samples, e.g., human serum samples without any norovirus-specific antibodies, were not available. However, a blank control consisting of human immunoglobulin depleted serum was added on every plate to monitor non-specific assay reactions. Low and high positive control samples were collected from healthy volunteers of which the antibody responses were investigated prior to the initiation of clinical testing. All serum donors have given written informed consent for the general use of their serum for the development of quality control samples and standards and the development of assays.

### Cross-reactive IgG antibodies against non-vaccine VLPs

ELISA for the end-point titration analysis of NoV-specific IgG antibodies present in human serum was performed as described (34, 35). Responses to four different NoV genotypes [GI.3 (2002), GII.4 (1999), GII.4 Sydney (2012) and GII.17 Kawasaki 308 (2015)] were examined. The evolutionary relationships of VP1 protruding domains are shown in **Supplementary Figure 3**. Baculovirus-derived VLPs, produced and purified by Vaccine Development and Immunology (VDI) group at Vaccine Research Center (VRC), Tampere University, Finland were used as antigens for coating 96-well microtiter plates. The primary binding antibodies present in human sera recognizing these antigens are subsequently bound to the VLPs. These bound antibodies are detected by a secondary, peroxidase enzyme-labeled antibody used for determination of OD by a microplate absorbance reader. The titer is defined as the reciprocal of the highest serum dilution that gives a positive result.

### Histo-blood group antigen blocking assays

An ELISA format was used to determine NoV-specific serum antibodies, which are able to bind to NoV-derived VLPs and block their binding to cellular attachment factors, histo-blood group antigens (HBGAs), present in pig gastric mucin (PGM) (34, 36). The assay was optimized for measuring homologous blocking antibodies against NoV VLPs, GI.4 Chiba 407 (1987) and Gll.4 Aomori 2 (2006), used as vaccine strains.

NoV VLPs are pre-incubated with serially diluted human serum samples (primary antibodies) and then plated on 96-well microtiter plates coated with PGM HBGAs. Free VLPs, not blocked by the serum antibodies, are free to bind to HBGAs during incubation period. HBGA ligand-bound VLPs are detected by NoV-specific antibody (e.g, rabbit polyclonal anti-NoV antibody), followed by secondary antibody with covalently conjugated peroxidase enzyme label for detection (optical density, OD). The blocking capacity of NoV-specific serum antibodies is defined by comparing the VLP binding level in the presence of serum VLP pretreatment versus binding level in the absence of serum VLP pretreatment (maximum VLP binding) and expressed by blocking index percent (%). The blocking index (%) is calculated for each serum dilution using the following equation:


$$Blocking\;index\;(\%)=100\%-(\frac{Mean\;OD_{VLP\;pretreated}}{Mean\;OD_{MaxVLP\;binding\times 100\%}})$$

A sigmoidal curve generated from blocking indexes is used to determine the half maximal inhibitory concentration (IC50) value for each serum sample, which represents the effective concentration (titer) of the serum where the blocking of the VLP binding is reduced by half of the maximal VLP blocking.

### Cell-mediated immune response - Lymphoproliferation assay

PBMC were thawed and suspended in RPMI 1640 medium supplemented with Minimum Essential Medium non-essential amino acids, L-glutamine, penicillin/streptomycin, sodium pyruvate, 2-mercapto-ethanol (all from Invitrogen) and heat inactivated AB serum (Valley Biomedical). This ‘complemented’ RPMI 1640 medium is hereafter termed ‘complete medium’. During the assay, PBMC were tested in triplicate and either kept in complete medium only (unstimulated control cultures) or stimulated with 2 µg/mL of antigens for 5 days at +37°C in an atmosphere of 5% CO_2_ in air, at which time 10 µCi [^3^H]-thymidine (Perkin Elmer) was added to each microculture. After 16 to 20 h at +37°C and 5% CO_2_, the cultures were harvested onto glass-fiber filters (Perkin-Elmer) using a multichannel cell harvester (Inotech) and incorporation of [^3^H]-thymidine was measured by liquid scintillation counting (MicroBeta counter Trilux from Wallac). The antigens used for lymphocyte stimulation were the vaccine antigens GI.4 Chiba 407 (1987) and GII.4 Aomori 2 (2006). Results are expressed as stimulation index (SI = mean counts per minute of antigen-stimulated cultures/mean counts per minute of control cultures).

### Statistical analysis

The study was not powered for any statistical hypothesis testing. Descriptive statistics were used to summarize all relevant parameters: number and percentage for discrete variables and mean (arithmetic or geometric), median, standard deviation (SD), interquartile range (IQR), 95% confidence interval (CI), minimum (min), and maximum (max) for continuous variables.

In general, data are presented for each study group, with placebo participants from both cohorts (dose groups) combined into a single, overall placebo group. Data for all study subjects combined are also presented when appropriate.

The exact two-sided 95% confidence intervals (CIs) for a proportion within a group were calculated using the Clopper-Pearson exact method. The geometric mean titers (GMTs) calculations were performed by taking the anti-log of the mean of the log10 titer transformation. Confidence intervals for geometric means were derived by raising 10 to the CI associated with the mean of the log10 values i.e., CI of geometric mean = 10^(CI for the mean of the log10 values). All subjects in the analysis set of interest with data were considered. Subjects whose antibody titers were below the cut-off of the assay were given an arbitrary value of half the cut-off for the purpose of GMT calculation (i.e., if the titer value is reported as “<X”, X/2 was used). Groups were compared using a mixed model with a Satterthwaite denominator-degrees-of-freedom approximation. Results were considered to be significant when the p value was <0.001.

All derivations, statistical analyses, summaries, and listings were generated using SAS version 9.4 or higher (SAS Institute, Inc., Cary, North Carolina).

## Results

### Study population

**Figure 2:**
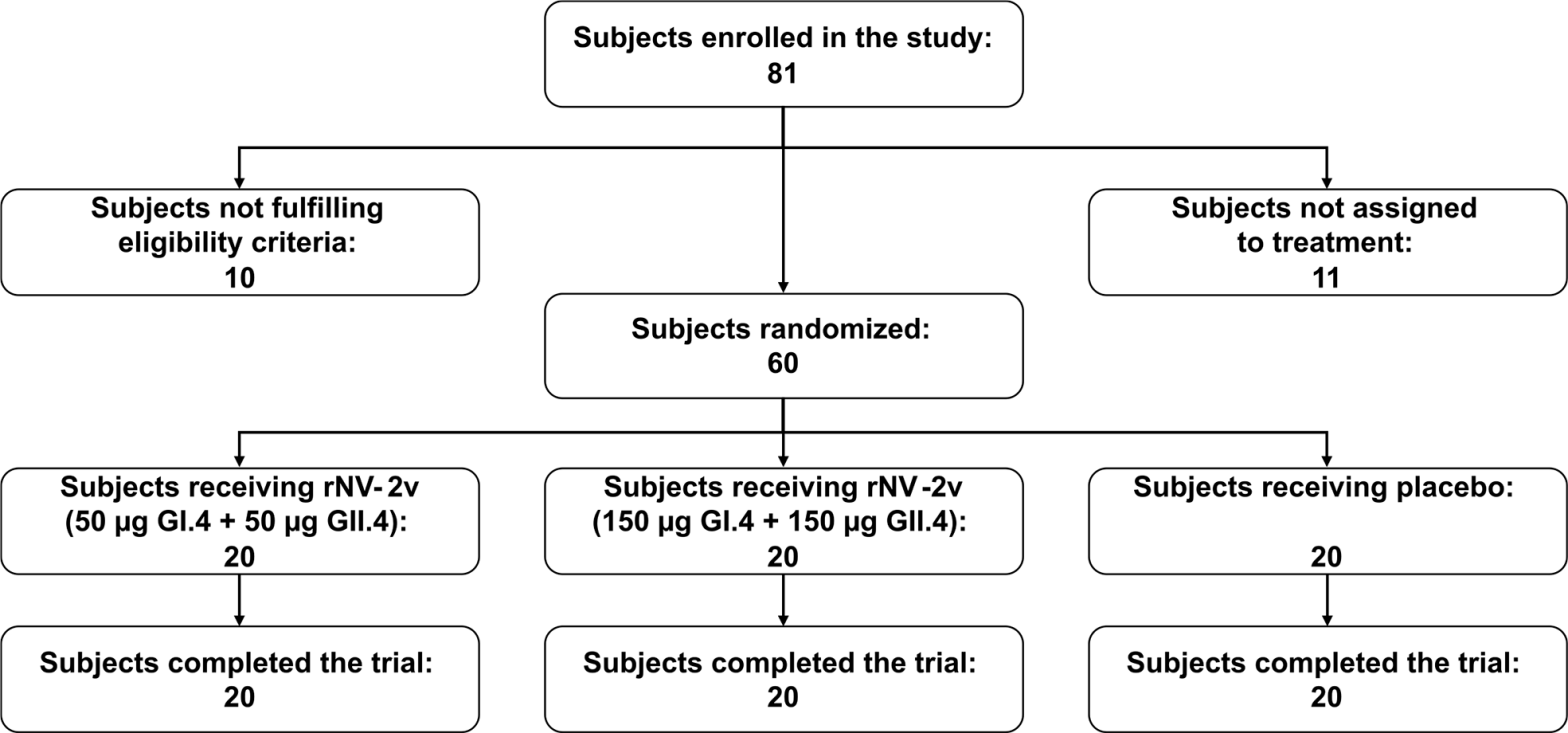
Subject disposition.

Of 81 subjects screened, 71 were found eligible and 60 of them were randomized. All randomized subjects completed the study and were included in both the safety and immunogenicity analysis sets (Subject disposition - **Figure 2**). Demographic and baseline characteristics of the participants, most of whom were female (48 subjects [80%]), are shown in **Table 1**. The three study groups, rNV-2v 50 µg GI.4 + 50 µg GII.4 (rNV-2v 50 µg), rNV-2v 150 µg GI.4 + 150 µg GII.4 (rNV-2v 150 µg), and placebo (vehicle) were similar in most demographic and baseline characteristics. All subjects received the 2 doses of the investigational medicinal product and completed the study.

**Table 1:** Demographics and baseline characteristics of participants at enrolment.

Characteristic	rNV-2v[50 ug GI.4+ 50 ug GII.4](N=20)	rNV-2v[150 ug GI.4+ 150 ug GII.4](N=20)	Placebo(N=20)	All Subjects(N=60)
Sex				
Male n (%)	4 (20.0)	5 (25.0)	3 (15.0)	12 (20.0)
Female n (%)	16 (80.0)	15 (75.0)	17 (85.0)	48 (80.0)
Race				
White - Caucasian n (%)	20 (100.0)	20 (100.0)	18 (90.0)	58 (96.7)
White - Arabic n (%)	0	0	2 (10.0)	2 (3.3)
Age (years) mean (SD)	30.8 (6.9)	28.6 (6.2)	28.8 (6.9)	29.4 (6.6)
Age range (years)	19 - 39	21 - 40	19 - 40	19 - 40
Height (cm) mean (SD)Height range (cm)	171.6 (7.8)155 - 185	170.6 (7.8)161 - 186	168.9 (6.4)158 - 178	170.4 (7.3)155 - 186
Weight (kg) mean (SD)	73.1 (10.6)	72.4 (13.0)	65.4 (9.7)	70.3 (11.6)
Weight range (kg)	50 - 94	53 - 98	47 - 90	47 - 98
BMI (kg/m^2^) mean (SD)	24.78 (3.33)	24.94 (4.76)	22.83 (2.32)	24.18 (3.68)
BMI range (kg/m^2^)	19.3 - 33.3	18.8 - 36.9	18.8 - 29.1	18.8 - 36.9

BMI, body mass index; SD, standard deviation.

### Safety and tolerability

**Figure 3:**
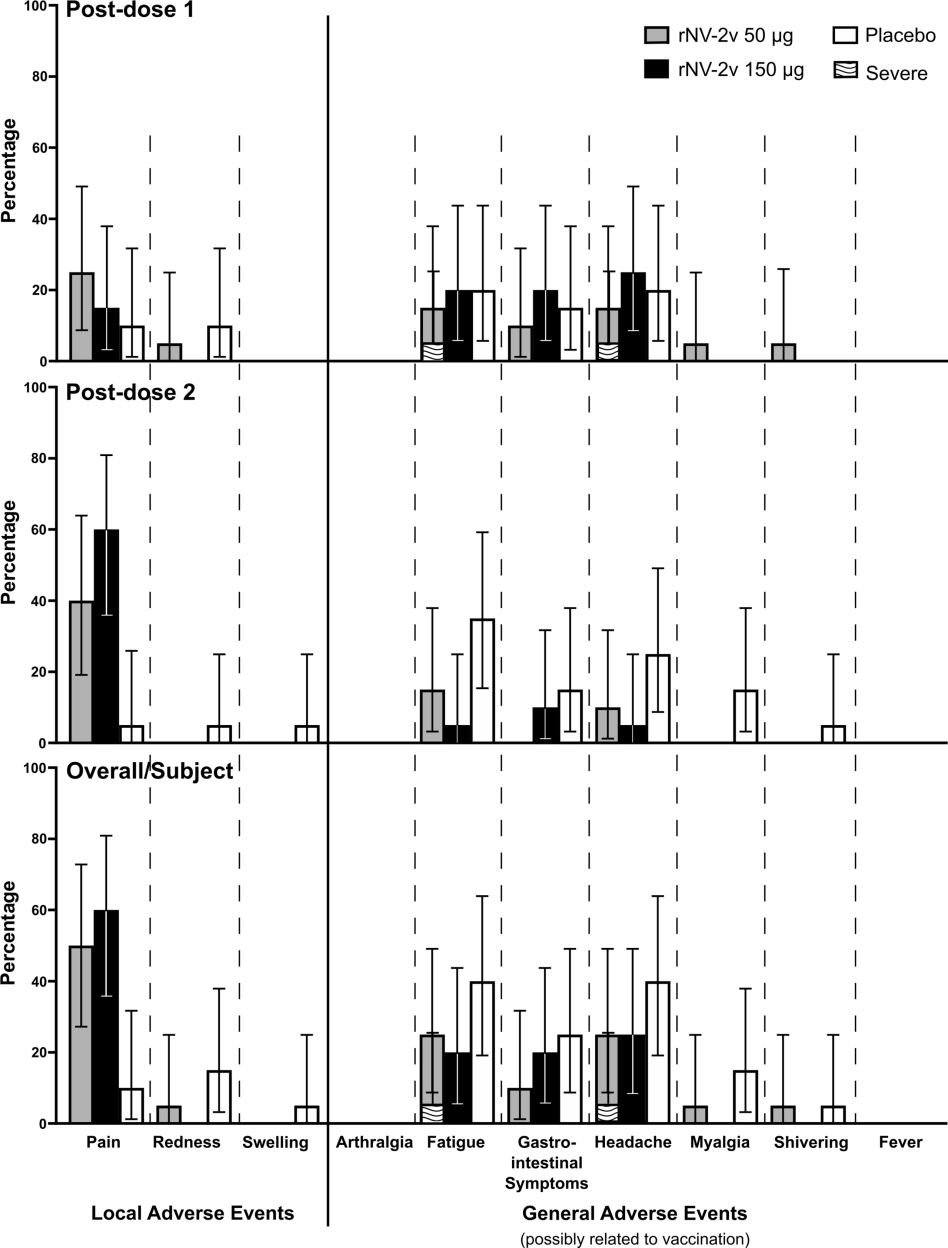
Solicited local and general adverse events during the 7-day post-vaccination period. Percentages of participants (with exact 95% confidence intervals) reporting solicited local adverse events (pain, redness and swelling) and general adverse events (arthralgia, fatigue, gastrointestinal symptoms, headache, myalgia, shivering and fever) possibly related to the vaccination during the 7-day post-vaccination period after each dose and overall per subject. Gastrointestinal symptoms defined as nausea, vomiting, diarrhea and/or abdominal pain. Fever defined as temperature ≥38.0°C. Error bars for zero values are omitted for the sake of clarity.

The primary objective of this Phase I study was to assess the frequency and severity of adverse events. Overall, 13 subjects (65.0%) in the rNV-2v 50 µg group, 16 subjects (80.0%) in the rNV-2v 150 µg group, and 14 subjects (70.0%) in the placebo group reported at least 1 solicited local or general symptom during the 7-day post-vaccination periods following each dose. **Figure 3** shows the percentages of participants reporting solicited local and general adverse events during the 7-day post-vaccination period after each dose and overall per subject. Overall, pain at the injection site occurred more frequently in the rNV-2v 50 µg group (10 subjects or 50.0%) and in the rNV-2v 150 µg group (12 subjects or 60.0%) than in the placebo group (2 subjects or 10.0%). No severe solicited local AEs or medically attended visits related to solicited local AEs were reported during the 7-day post-vaccination periods following each dose (see **Supplementary Table 3**). In the rNV-2v 50 µg group, overall, 6 subjects (30.0%) had fatigue (5 possibly related to vaccination, 25%), 4 subjects (20.0%) had gastrointestinal symptoms (2 possibly related to vaccination, 10%), 7 subjects (35.0%) had headache (5 possibly related to vaccination, 25%), 3 subjects (15.0%) had myalgia (1 possibly related to vaccination, 5%), and 2 subjects (10.0%) had shivering (1 possibly related to vaccination, 5%). In the rNV-2v 150 µg group, overall, 1 subject (5.0%) had arthralgia (unrelated to vaccination), 5 subjects (25.0%) had fatigue (4 possibly related to vaccination, 20%), 4 subjects (20.0%) had gastrointestinal symptoms (all possibly related to vaccination), 7 subjects (35.0%) had headache (5 possibly related to vaccination, 25%), and 1 subject (5.0%) had myalgia (unrelated to vaccination). In the placebo group, overall, 9 subjects (45.0%) had fatigue (8 possibly related to vaccination, 40%), 7 subjects (35.5%) had gastrointestinal symptoms (5 possibly related to vaccination, 25%), 10 subjects (50.0%) had a headache (8 possibly related to vaccination, 40%), 3 subjects (15.0%) had myalgia (all possibly related to vaccination), and 2 subjects (10.0%) had shivering (1 possibly related to vaccination, 5%). Severe solicited general AEs of fatigue (1 subject, 5.0%, experienced on two consecutive days) and headache (1 subject, 5.0%, experienced on two consecutive days) were reported in the rNV-2v 50 µg group during the 7-day post-vaccination periods following each dose (**Figure 3**; **Supplementary Table 4**).

A total of 8 subjects (40.0%) in each group (rNV-2v both 50 µg and 150 µg groups and placebo group) reported at least 1 unsolicited AE during the 28-day post-vaccination periods following each dose (see **Supplementary Table 5**). Unsolicited AEs considered to have a causal relationship with the vaccination were reported in 1 subject (5.0%) in the rNV-2v 50 µg group (muscle spasms), 1 subject (5.0%) in the rNV-2v 150 µg group (injection site pruritus), and 2 subjects (10.0%) in the placebo group (dizziness). No severe unsolicited AEs with causal relationship to vaccination during study and no serious adverse events were reported during this study.

Analyses of laboratory parameters, vital signs, and physical examination findings did not reveal any clinically relevant effects of the rNV-2v vaccine for either dose.

### Immunogenicity

#### Serum IgG antibodies against vaccine VLPs GI.4 and GII.4

**Figure 4:**
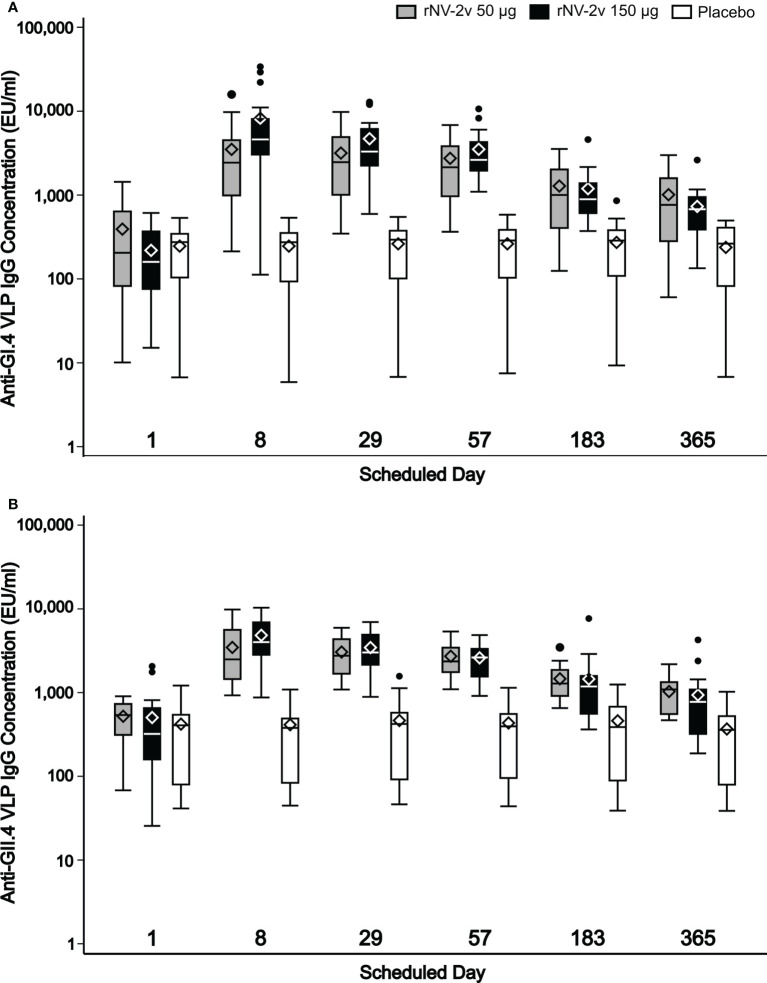
Humoral anti-GI.4 and anti-GII.4 VLP IgG responses. Humoral anti-GI.4 and anti-GII.4 VLP IgG responses by scheduled time for each treatment. OD values were processed using a 4-parameter logistic fitting algorithm to the standard curve, allowing the determination of antibody concentration in the samples, expressed as EU/mL. Anti-GI.4 **(A)** and anti-GII.4 **(B)**. VLP IgG responses for rNV-2v 50 µg (grey), 150 µg (black) and placebo (white) are shown as boxplots. The horizontal line represents the median, the bottom and top of the box represent 25^th^ and 75^th^ percentiles of the values, respectively, and the upper and lower error bar represent 5^th^ and 95^th^ percentile of the values, respectively. Diamond indicates mean value. Dots represent outliers. IgG, immunoglobulin G, VLP, virus-like particle.

The magnitude and kinetics of the humoral immune response against the vaccine antigens, GI.4 and GII.4 VLPs are shown in **Table 2** and **Figure 4**. Almost all subjects displayed anti-GI.4 and anti-GII.4 IgG at baseline (day 1) and no relevant differences in baseline levels were detected between the three groups. The baseline values for anti-GII.4 IgG were higher compared to baseline anti-GI.4 IgG for all three groups. The anti-GI.4 and anti-GII.4 titers increased after the first vaccination but did not rise further after the second vaccination in both rNV-2v groups. The geometric mean titers of anti-GI.4 and anti-GII.4 VLP IgG remained similar across visits in the placebo group. A trend of higher levels of IgG response was observed in the higher dose group (rNV-2v 150 µg group) compared to the lower dose group (rNV-2v 50 µg group) at early post-vaccination timepoints but the differences were not statistically significant at any timepoint. Antibody responses against both VLPs expressed as geometric mean titers and geometric mean fold-rise reached peak values on day 8 (150 µg groups) or day 29 (50 µg group) after the 1^st^ vaccine dose and decreased from day 29 or 57 onward. Antibody concentrations against both VLPs on day 365 still exceeded those measured at baseline (day 1) and placebo control at a statistically significant level (p<0.001). For both anti-GI.4 and anti-GII.4 VLP IgG responses, an inverse correlation was observed between the baseline IgG value and the fold-rise, but also in the high baseline group a fold-rise could still be elicited.

**Table 2:** Antibody response to vaccine antigens expressed as Geometric Mean Titer (EU/mL) and Geometric Mean Fold Rise of anti-GI.4 and anti-GII.4 VLP IgG at scheduled time points for each study group.

			Time point					
Assay	Study group	Parameter 95% CI	Day 1	Day 8	Day 29	Day 57	Day 183	Day 365
Anti-GI.4	rNV-2v/50	Geomean* 95% CI	164.1 182.8-601.3	2126.6 1740.9-5264.9	2132.2 1887.5-4439.1	2018.0 1752.7-3722.4	867.7 800.2-1760.5	612.2 598.2-1423.2
		GMFR 95% CI	ND ND	13.0 15.1-39.6	13.0 14.2-35.1	12.3 12.4-37.6	5.3 4.8-11.8	3.7 3.1-7.7
	rNV-2v/150	Geomean 95% CI	141.0 131.4-306.6	4762.0 3883.4-12539.5	3544.4 2941.8-6444.3	2926.2 2368.8-4655.3	974.0 748.9-16534.6	581.5 477.3-987.1
		GMFR 95% CI	ND ND	33.8 29.7-93.1	25.1 21.7-59.7	20.8 17.8-50.1	6.9 6.0-13.2	4.1 3.5-7.0
	Placebo	Geomean 95% CI	160.1 169.2-323.3	158.4 168.9-324.0	167.4 179.4-344.7	168.7 177.6-347.2	173.1 173.8-371.8	149.2 157.1-318.4
		GMFR 95% CI	ND ND	1.0 1.0-1.0	1.0 1.0-1.1	1.1 1.0-1.1	1.1 1.0-1.2	0.9 0.9-1.0
Anti-GII.4	rNV-2v/50	Geomean 95% CI	440.0 401.0-641.9	2665.3 2206.9-4720.3	2665.6 2300.9-3802.6	2437.6 2103.9-3328.2	1319.2 1128.9-1797.6	927.5 807.1-1254.4
		GMFR 95% CI	ND ND	6.1 5.4-18.6	6.0 2.8-19.6	5.5 1.9-17.7	3.0 1.3-7.6	2.1 1.2-4.4
	rNV-2v/150	Geomean 95% CI	286.8 249.0-758.0	3972.4 3517.8-6142.4	2969.7 2626.5-4275.4	2313.7 2036.6-3161.0	1040.8 697.5-2203.6	650.5 489.1-1382.4
		GMFR 95% CI	ND ND	13.9 6.3-54.0	10.4 5.1-34.2	8.1 5.8-19.5	3.6 2.7-6.9	2.3 1.8-3.5
	Placebo	Geomean 95% CI	266.3 247.6-593.2	256.0 236.5-588.5	281.3 256.3-672.1	273.1 253.0-613.6	290.6 270.5-651.0	231.1 211.0-524.2
		GMFR 95% CI	ND ND	1.0 0.9-1.0	1.1 1.0-1.2	1.0 1.0-1.1	1.1 0.9-1.3	0.9 0.9-1.0

CI, confidence interval; Geomean, geometric mean antibody concentration; GMFR, geometric mean fold-rise of the specific timepoint versus geomean at day 1 (= pre-vaccination); ND, not determined.

*Geometric mean values are expressed as EU/mL (ELISA units per millilitre).

### Cross-reactive serum antibodies against non-vaccine VLP of genotypes of GI and GII

**Figure 5:**
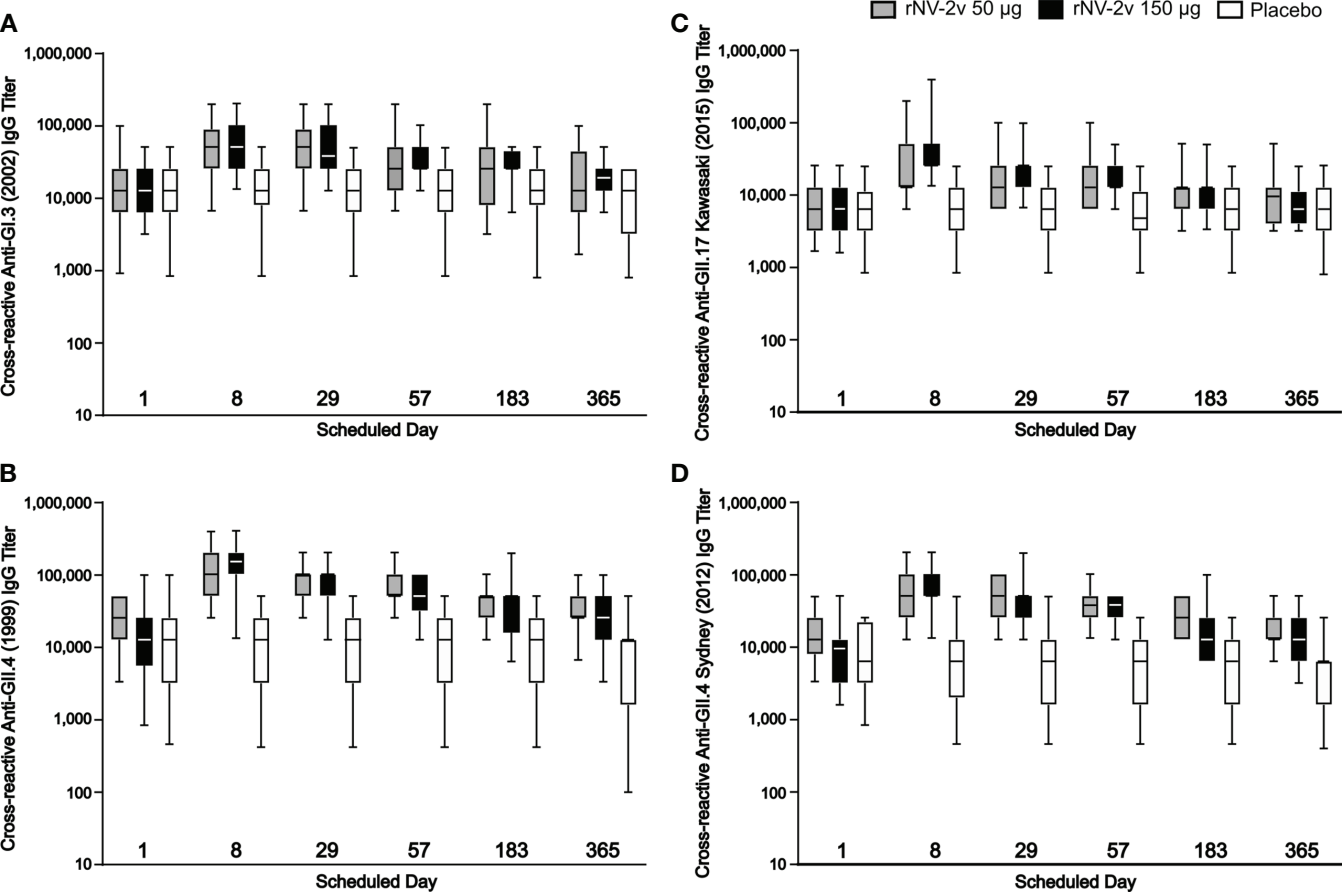
Humoral, cross-reactive anti-GI.3, anti-GII.4 and anti-GII.17 VLP IgG responses. Humoral, cross-reactive IgG responses against non-vaccine strain VLP scheduled time for each treatment. The cross-reactive IgG titer is defined as the reciprocal of the highest serum dilution that gives a positive result. Anti-GI.3 (2002) **(A)**, anti-GII.4 (1999) **(B)**, anti-GII.17 Kawasaki 308 (2015) **(C)** and anti-GII.4 Sydney (2012) **(D)** VLP IgG responses for rNV-2v 50 µg (grey), 150 µg (black) and placebo (white) are shown as boxplots. The horizontal line represents the median, the bottom and top of the box represent 25^th^ and 75^th^ percentiles of the values, respectively, and the upper and lower error bar represent 5^th^ and 95^th^ percentile of the values, respectively. IgG, immunoglobulin G, VLP virus-like particle.

The rNV-2v vaccine antigens, GI.4 Chiba 407 (1987) VP1 and GII.4 Aomori 2 (2006) VP1, elicited cross-reactive IgG antibodies against non-vaccine VLPs as shown in **Figure 5** and **Table 3**. These responses were more pronounced for the strains of the same genotype (closely related VP1 sequences) as the GII.4 vaccine strain, like GII.4 (1999) and GII.4 Sydney (2012) and less for non-vaccine genotypes such as GI.3 (2002) and GII.17 Kawasaki 308 (2015) (see **Supplementary Figure 1** for evolutionary relationships of VP1 protruding domains of vaccine and non-vaccine strains analyzed). Higher levels of cross reactivity were seen in the higher dose group (rNV-2v 150 µg) compared with the lower dose group (rNV-2v 50 µg) but these differences were not statistically significant at any timepoint post-vaccination. Accordingly, at all post-vaccination timepoints, the differences in least square (LS) adjusted geometric mean cross-reactive IgG titers between the rNV-2v 50 µg versus placebo group and rNV-2v 150 µg versus placebo group were statistically significant (p<0.001) for GII.4 (1999) and GII.4 Sydney (2012), whereas, for GII.17 Kawasaki 308 (2015) this was the case up to day 57. For GI.3 (2002) differences in least square (LS) adjusted geometric mean cross-reactive IgG titers between rNV-2v 50 µg versus placebo group were statistically significant (p<0.001) up to day 57 and for rNV-2v 150 µg versus placebo at all post-vaccination timepoints.

**Table 3:** Cross-reactive IgG antibody responses to non-vaccine antigens expressed as Geometric Mean Titres at scheduled time points.

Assay	Study group	Parameter95% CI	Time point					
			Day 1	Day 8	Day 29	Day 57	Day 183	Day 365
Anti-GI.3 (2002)	rNV-2v/50	Geomean*	13718.7	43053.9	40170.7	31517.3	20793.7	14703.3
		95% CI	11593.5-34566.5	35604.8-78955.2	33193.7-77526.3	24045.6-67474.4	15273.9-58966.1	13046.4-36713.6
		GMFR	ND	3.1	2.9	2.3	1.5	1.1
		95% CI	ND	2.9-5.7	2.3-6.5	1.5-5.6	0.7-3.9	0.7-2.3
	rNV-2v/150	Geomean	13718.7	54874.8	41587.3	30443.7	25600.0	18740.3
		95% CI	11528.6-26231.4	45386.2-104373.8	34171.3-79748.7	24843.0-49397.0	22749.4-36130.6	15773.2-29026.8
		GMFR	ND	4.0	3.0	2.2	1.9	1.4
		95% CI	ND	2.7-11.3	2.3-6.4	1.8-3.8	1.4-3.4	1.2-1.9
	Placebo	Geomean	11942.8	11942.8	10042.7	10042.7	11143.0	8887.4
		95% CI	11400.4-26599.6	11177.7-26182.3	9314.7-21005.3	9171.6-20508.4	10684.3-24035.7	8436.4-17079.4
		GMFR	ND	1.0	0.8	0.8	0.9	0.8
		95% CI	ND	0.9-1.1	0.7-1.1	0.7-1.1	0.7-1.5	0.7-1.0
Anti-GII.4 (1999)	rNV-2v/50	Geomean	20085.4	98911.9	82269.3	69941.3	41587.3	32628.8
		95% CI	17985.7-34814.3	83849.8-172150.2	68624.1-128091.6	57520.6-111439.4	35783.7-64056.3	28146.9-48013.1
		GMFR	ND	4.9	4.1	3.5	2.1	1.6
		95% CI	ND	4.3-13.1	2.9-9.9	2.2-8.6	1.3-4.4	0.8-3.9
	rNV-2v/150	Geomean	11536.0	126069.2	77604.7	51200.0	33779.4	23885.6
		95% CI	10213.4-31626.6	113386.3-213013.7	70582.5-129097.5	46813.7-79906.3	27229.5-70050.5	21711.4-44528.6
		GMFR	ND	10.9	6.7	4.4	2.9	2.1
		95% CI	ND	6.2-33.5	4.0-17.6	3.3-10.1	2.4-4.8	1.7-3.2
	Placebo	Geomean	9700.6	9370.1	8444.9	7879.3	8157.2	5531.0
		95% CI	8839.0-31841.0	10333.1-26426.9	9380.7-25619.3	8392.9-24687.1	8568.3-22911.7	5690.3-18067.6
		GMFR	ND	1.0	0.9	0.8	0.8	0.6
		95% CI	ND	0.9-1.1	0.8-1.0	0.7-1.0	0.7-1.1	0.6-0.8
Anti-GII.4 Sydney (2012)	rNV-2v/50	Geomean	14202.5	53005.6	44572.2	40170.7	25600.0	17485.3
		95% CI	12128.9-22751.1	46021.6-99898.4	39000.9-71079.1	33734.0-62266.0	22141.8-36738.2	14600.8-26359.2
		GMFR	ND	3.7	3.1	2.8	1.8	1.2
		95% CI	ND	3.2-10.6	2.1-8.9	1.5-7.9	1.0-4.1	0.9-2.5
	rNV-2v/150	Geomean	7610.9	67558.8	43053.9	31517.3	16314.4	11143.0
		95% CI	6040.9-19559.1	57477.4-112762.6	34097.7-75982.3	28163.4-43516.6	12244.7-33835.3	9267.2-22732.8
		GMFR	ND	8.9	5.7	4.1	2.1	1.5
		95% CI	ND	7.1-25.0	3.4-17.1	3.6-7.7	1.9-3.1	1.3-1.8
	Placebo	Geomean	6859.4	5768.0	5198.4	4685.1	5198.4	4608.8
		95% CI	6232.8-15127.2	4841.5-16638.5	3997.2-15402.8	4217.8-11182.2	4715.9-12444.1	3736.1-10790.3
		GMFR	ND	0.8	0.8	0.7	0.8	0.7
		95% CI	ND	0.7-1.1	0.7-1.0	0.6-0.8	0.7-1.0	0.6-1.1
Anti-GII.17 Kawasaki 308 (2015)	rNV-2v/50	Geomean	6859.4	21526.9	16314.4	15758.6	10763.5	9370.1
		95% CI	5564.9-12835.1	14596.1-58363.9	12426.4-34933.6	11556.7-33243.3	8211.8-21228.2	7199.9-20640.1
		GMFR	ND	3.1	2.4	2.3	1.6	1.4
		95% CI	ND	2.0-8.8	1.7-4.9	1.6-4.8	1.2-2.8	1.0-2.5
	rNV-2v/150	Geomean	6400.0	40170.7	20085.4	16314.4	10763.5	6625.7
		95% CI	5248.3-12671.7	19297.5-99742.5	14787.1-33212.9	13710.7-23409.3	8270.9-18289.1	5339.9-10340.1
		GMFR	ND	6.3	3.1	2.5	1.7	1.0
		95% CI	ND	4.0-18.2	2.6-6.1	1.8-5.1	1.4-3.1	0.9-1.7
	Placebo	Geomean	5381.7	5571.5	5198.4	4850.3	5768.0	5332.9
		95% CI	4450.6-9869.4	4791.3-10328.7	4402.9-10077.1	3962.2-9557.8	4906.2-10373.8	4487.9-10417.3
		GMFR	ND	1.0	1.0	0.9	1.1	1.0
		95% CI	ND	0.9-1.2	0.9-1.1	0.8-1.0	0.9-1.4	0.5-2.1

CI, confidence interval; Geomean, geometric mean antibody concentration; GMFR, geometric mean fold-rise of the specific timepoint versus geomean at day 1 (= pre-vaccination); ND, not determined.

*Geometric mean values are expressed as titer.

### Serum antibodies block the binding of GI.4 and GII.4 VLPs to histo-blood group antigen

**Figure 6:**
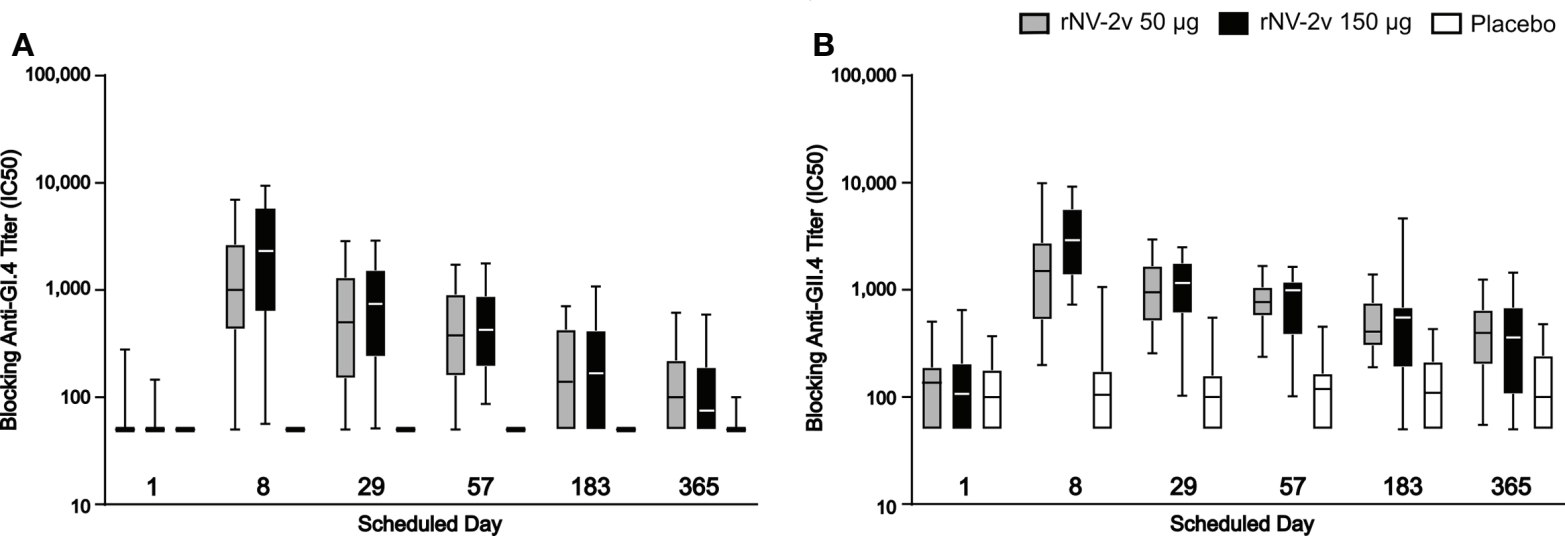
Serum antibodies blocking GI.4 and GII.4 VLP binding to histo-blood group antigen. Blocking of binding of GI.4 VLP **(A)** and GII.4 VLP **(B)** to Pig Gastric Mucin by antibodies elicited by rNV-2v 50µg (gray), 150 µg (black) and placebo (white) expressed as half maximal inhibitory concentration (IC50) which represents the effective concentration (titer) of the serum where the blocking of the VLP binding is reduced by half of the maximal VLP blocking. The horizontal line represents the median, the bottom and top of the box represent 25^th^ and 75^th^ percentiles of the values, respectively, and the upper and lower error bar represent 5^th^ and 95^th^ percentile of the values, respectively. PGM, Pig Gastric Mucin, VLP, virus-like particle.

As shown in **Figure 6** and **Table 4**, the levels of serum antibodies blocking the binding of GI.4 and GII.4 VLPs to histo-blood group antigen mirrored the kinetics and changes observed in the ELISAs. In both the rNV-2v 50 µg and 150 µg groups, the geometric mean of blocking anti-GI.4 and anti-GII.4 titers increased from day 1 to day 8 and decreased from day 29 to day 365. On day 8, the geometric mean of blocking anti-GI.4 and anti-GII.4 titers were higher in the rNV-2v 150 µg group compared to rNV-2v 50 µg group but the differences in LS adjusted geometric mean of blocking IgG titers between the two rNV-2v groups were not statistically significant at any timepoint.

**Table 4:** Serum antibodies blocking GI.4 and GII.4 VLP binding to histo-blood group antigen at scheduled time points for each study group.

			Time point					
Blocking Assay	Study group	Parameter95% CI	Day 1	Day 8	Day 29	Day 57	Day 183	Day 365
Anti-GI.4	rNV-2v/50	Geomean* 95% CI	62.3 45.6-100.6	837.0 843.7-2580.6	414.1 406.2-1200.9	349.1 331.6-866.2	152.1 136.0-351.9	109.9 83.4-247.1
		GMFR 95% CI	ND ND	13.4 14.1-50.0	6.6 5.5-20.7	5.6 4.7-14.0	2.4 2.0-5.3	1.8 1.3-3.3
	rNV-2v/150	Geomean 95% CI	52.8 44.5-65.6	1627.2 1678.4-4432.4	569.7 567.1-1411.6	422.1 371.6-830.8	170.7 141.4-389.1	94.6 68.0-205.7
		GMFR 95% CI	ND ND	30.8 32.1-87.9	10.8 10.5-27.6	8.0 6.9-16.2	3.2 2.6-7.5	1.8 1.4-3.3
	Placebo	Geomean 95% CI	50.0 ND	50.0 ND	50.0 ND	50.0 ND	50.0 ND	51.9 47.1-58.2
		GMFR 95% CI	ND ND	1.0 ND	1.0 ND	1.0 ND	1.0 ND	1.0 0.9-1.2
Anti-GII.4	rNV-2v/50	Geomean 95% CI	115.4 94.6-200.5	1360.3 1088.6-3343.8	904.6 786.9-1497.1	744.6 652.8-1023.0	472.0 396.7-715.2	362.2 317.9-602.6
		GMFR 95% CI	ND ND	11.8 9.5-40.8	7.8 6.7-17.0	6.5 5.7-11.4	4.1 3.4-6.7	3.1 2.7-5.4
	rNV-2v/150	Geomean 95% CI	121.3 96.8-260.6	2764.5 2432.6-4731.0	871.4 844.9-1520.7	703.4 657.3-1099.8	408.5 233.5-1186.8	286.2 262.0-637.1
		GMFR 95% CI	ND ND	22.8 16.7-62.8	7.2 5.3-16.4	5.8 4.7-10.1	3.4 2.8-5.8	2.4 1.9-3.6
	Placebo	Geomean 95% CI	102.4 84.5-177.7	107.5 55.2-273.1	101.5 76.0-196.0	104.9 83.8-187.6	113.6 96.8-203.	114.2 94.2-225.4
		GMFR 95% CI	ND ND	1.0 0.9-1.3	1.0 0.9-1.1	1.0 1.0-1.1	1.1 1.0-1.4	1.2 1.0-1.5

CI, confidence interval; Geomean, geometric mean antibody titre; GMFR, geometric mean fold-rise of the specific timepoint versus geomean at day 1 (= pre-vaccination); ND, not determined.

*Geometric mean values are expressed as half maximal inhibitory concentration/titer (IC50).

In the placebo group, the geometric mean of blocking anti-GI.4 titers remained similar across visits. On days 8, 29, and 57, the differences in LS adjusted geometric mean of blocking anti-GI.4 titers between the rNV-2v 50 µg vs. placebo group were statistically significant (p<0.001). On days 8, 29, 57, and 183, the differences in LS adjusted geometric mean of blocking anti-GI.4 titers between the rNV-2v 150 µg vs. placebo group were statistically significant (p<0.001). In both the rNV-2v 50 µg and rNV-2v 150 µg groups and at all-time points the blocking anti-GII.4 titers were significantly higher (p<0.001) than in the placebo group.

### Cellular immune response measured as GI.4- and GII.4-specific lymphoproliferative responses

**Figure 7:**
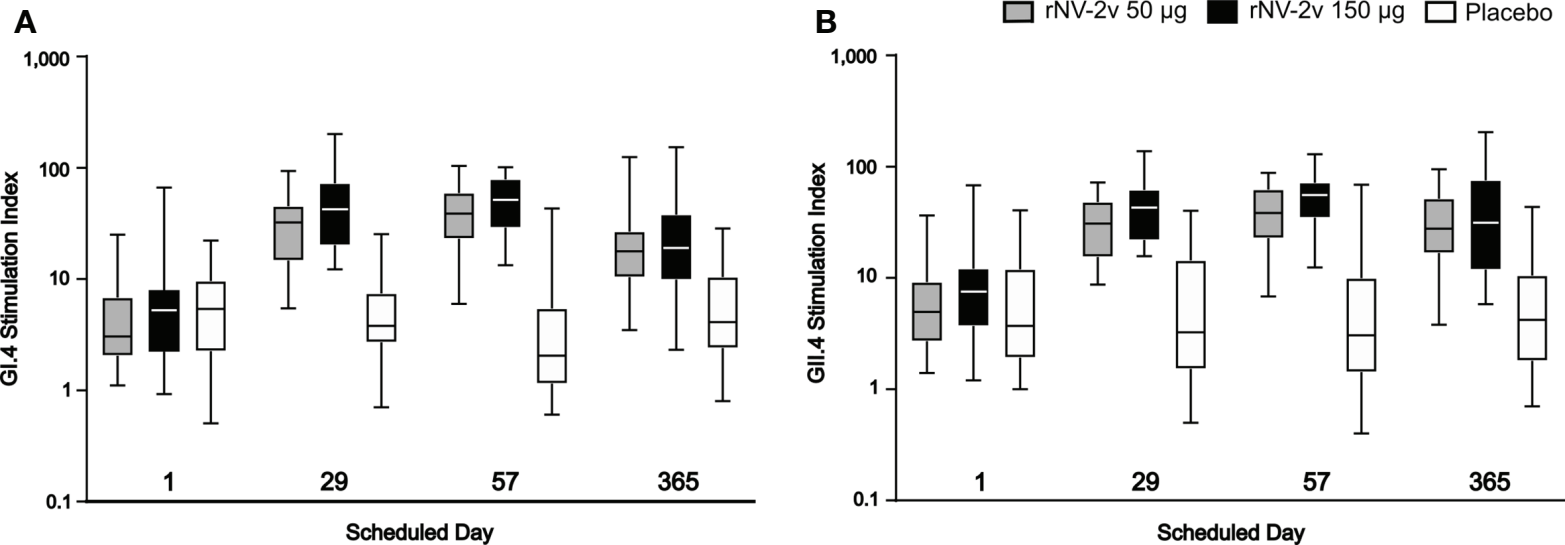
Cellular immune response. Cellular immune response measured as GI.4 and GII.4 VLP-specific lymphoproliferative responses by scheduled time for each study group. GI.4 **(A)** and GII.4 **(B)** VLP-specific responses for rNV-2v 50µg (grey), 150 µg (black) and placebo (white) are expressed as stimulation indices and shown as boxplots. The horizontal line represents the median, the bottom and top of the box represent 25^th^ and 75^th^ percentiles of the values, respectively, and the upper and lower error bar represent 5^th^ and 95^th^ percentile of the values, respectively. VLP, virus-like particle.

Most subjects displayed lymphoproliferative responses (Stimulation Index, SI ≥ 2) at baseline (day 1), namely 48 of 60 for GI.4 and 51 of 60 for GII.4. In both rNV-2v dose groups, the geometric mean lymphoproliferative responses (SI) against GI.4 and GII.4 increased from day 1 to day 57 and decreased only slightly over time (day 365) (**Table 5** and **Figure 7**). In the placebo group, the lymphoproliferative responses (SI) remained similar across visits. At all post-vaccination timepoints, the differences in LS adjusted geometric means of GI.4 and GII.4 SI between the rNV-2v 50 µg vs. placebo group and rNV-2v 150 µg vs. placebo group were statistically significant (p<0.001). The differences in LS adjusted geometric mean lymphoproliferative responses (SI) for GI.4 and GII.4 between the two 50 µg and 150 µg NV-2v groups were not statistically significant (p>0.001) at any timepoint post-vaccination. As for the anti-GI.4 and anti-GII.4 VLP IgG responses, an inverse correlation was observed between the baseline (day 1) SI’s and those measured at day 8 and day 57.

**Table 5:** Lymphoproliferation GI.4 and GII.4 Stimulation Index (SI) at scheduled time points for each treatment.

			Time point			
LPA	Study group	Parameter95% CI	Day 1	Day 29	Day 57	Day 365
GI.4	rNV-2v/50	Geomean*	3.53	24.96	34.15	19.07
		95% CI	2.36-7.48	21.92-42.61	30.00-51.91	14.39-44.71
		GMFR	ND	7.1	9.7	5.4
		95% CI	ND	6.4-15.8	8.0-19.7	4.7-13.8
	rNV-2v/150	Geomean	4.74	38.72	45.05	18.36
		95% CI	1.62-15.41	30.35-72.04	40.17-66.34	12.47-43.89
		GMFR	ND	8.2	9.5	3.9
		95% CI	ND	7.2-20.4	7.8-22.9	3.0-15.3
	Placebo	Geomean	4.42	4.12	2.61	4.29
		95% CI	3.97-9.65	3.30-8.98	0.55-9.53	3.37-10.20
		GMFR	ND	0.9	0.6	1.0
		95% CI	ND	0.8-1.3	0.5-1.1	0.7-2.9
GII.4	rNV-2v/50	Geomean	5.40	28.27	33.99	25.44
		95% CI	3.86-11.88	24.80-42.64	30.72-53.78	22.54-45.99
		GMFR	ND	5.2	6.3	4.7
		95% CI	ND	4.2-11.6	4.4-15.2	3.3-14.5
	rNV-2v/150	Geomean	7.44	39.22	49.87	29.19
		95% CI	4.97-20.38	32.92-59.45	43.39-75.33	23.58-69.29
		GMFR	ND	5.3	6.7	3.9
		95% CI	ND	4.5-11.7	5.2-19.8	3.4-9.4
	Placebo	Geomean	4.72	3.88	3.62	4.49
		95% CI	3.67-14.25	3.10-12.06	1.21-15.50	3.06-13.34
		GMFR	ND	0.8	0.8	1.1
		95% CI	ND	0.7-1.2	0.4-2.4	0.6-2.6

CI, confidence interval; Geomean, geometric mean; GMFR, geometric mean fold-rise of the specific timepoint versus geomean at day 1 (= pre-vaccination); ND, not determined; LPA, Lymphoproliferation assay.

Geometric mean values are expressed as stimulation index (SI).

## Discussion

This is the first clinical study to test the safety and immunogenicity of rNV-2v, a plant-produced, bivalent (GI.4 and GII.4) norovirus vaccine candidate consisting of non-adjuvanted VLPs. The trial demonstrated that two consecutive administrations of the candidate vaccine at two dose levels (50 µg + 50 µg and 150 µg + 150 µg) were safe and well tolerated. The first dose of each formulation elicited strong and broadly binding and blocking antibody responses to vaccine and non-vaccine strain VLPs and cellular immune responses to vaccine VLPs. These responses were not further boosted by the second dose.

Efficient *in planta* manufacturing allowed high yield production of the antigens and formulation of the vaccine candidate without adjuvant at a 1:1 ratio of 50 µg and 150 µg of each of the antigens. The formulation showed high stability at refrigerator temperature (+5 ± 3°C) for over one year (**Supplementary Table 6**). The safety and reactogenicity profile of rNV-2v in the tested population was comparable to that of an alum-adjuvanted, recombinant protein norovirus vaccine candidate that was also administered intramuscularly (16, 17, 19).

As a consequence of natural exposure to noroviruses, most study participants had detectable serum antibodies and displayed cellular immune responses to the vaccine VLPs at baseline. The candidate vaccine elicited strong increases of GI.4 and GII.4 binding and blocking antibodies and antigen-specific lymphoproliferative responses that peaked 8 to 28 days after the administration of the first dose. No significant differences in the magnitude of these responses were observed between the 50 µg and 150 µg dose levels and no booster effect was seen following the second dose. The fold increases of antibody levels and lymphoproliferative responses were inversely correlated with the baseline values of each of these parameters. The vaccine-induced humoral and cellular responses decreased with time but were still significantly higher 12 months after the first dose than at baseline (day 1).

The observation that 50 µg rNV-2v and 150 µg rNV-2v elicited comparable immune responses that already peaked 7 days after the first dose and the absence of boosting effect of a second dose can most likely be attributed to the priming effect of past norovirus infection(s). Further studies are needed to examine if a single dose of 50 µg will generate similar responses in young children (< 2 years of age) that are less likely to have been frequently exposed to the virus (35) or in older adults that are known to react less vigorously to vaccines due to immunosenescence (37). Since no difference in reactogenicity was observed between the 50 µg and 150 µg dose levels, there is ample margin to increase the vaccine dose if needed.

Serum antibodies that block the attachment of VLPs to HBGAs have also been quantified as the levels of these antibodies are associated with protection against infection and disease (38, 39). Vaccine induced changes in HBGA-blocking antibodies paralleled those observed for binding IgG antibodies measured by ELISA. HBGA-blocking titers of ≥200 have been reported to be associated with significant protection (38). This level is reached in the 50 µg and 150 µg groups for both VLPs but is less persistent for anti-GI.4 (day 57) than for anti-GII.4 (day 365).

In addition to raising the serum levels of binding and blocking antibodies against the vaccine strains GI.4 and GII.4, the candidate rNV-2v vaccine also elicits cross-reactive antibodies to non-vaccine GII.4 strains, like GII.4 (1999) and GII.4 Sydney (2012) and to a lesser extent to non-vaccine genotypes such as GI.3 (2002) and GII.17 Kawasaki 308 (2015). However, with respect to norovirus epidemiology and frequent changes in dominant strains and high diversity of circulating strains (40, 41) a vaccine providing a broad cross-protection is desirable (15). Therefore, future studies will evaluate vaccine candidate compositions with more than two antigens to increase protective capacity against the diverse norovirus strains that are already circulating and new ones that may arise.

The cellular immune response to vaccine VLPs was assessed using the lymphoproliferation assay. Most participants displayed a positive response (SI≥2) at baseline that increased significantly 4 weeks after the first dose. Here also, no further boosting effect of a second dose was noted. The observed cellular and humoral immune responses showed a clear correlation. The cell-mediated immunity will be examined in more detail using intracellular cytokine staining/flow cytometry. The results of these analyses will be presented in a separate publication.

Although comparisons of responses elicited by different vaccines studied in different clinical trials involving distinct participant populations and applying divergent immune monitoring assays, should be interpreted with caution, the similarity of safety and immunogenicity profiles of the present non-adjuvanted vaccine candidate and the alum-adjuvanted bivalent norovirus vaccine (TAK-214/HIL-214) developed by Takeda/HilleVax (17, 18, 20, 22) is striking. In this respect, it is especially noteworthy that the rNV-2v vaccine contains no adjuvants.

## Conclusion

In summary, Icon Genetics’ non-adjuvanted, bivalent VLP norovirus vaccine candidate was safe, well tolerated and immunogenic in young adults who showed signs of pre-existing immunity to the vaccine antigens GI.4 and GII.4. The data show that a single dose of the vaccine formulated at 50 µg of each VLP is sufficient to reach a peak response after 8 to 28 days. The results of this Phase I study certainly warrant further evaluation of this vaccine. Larger studies in children and/or older adults, the populations most at risk of developing severe norovirus gastroenteritis are needed to examine if these target groups respond equally well to a single dose of the non-adjuvanted rNV-2v vaccine. Higher valency vaccines will be evaluated as these may be required to achieve a broader cross-protection against norovirus strains that are circulating today and may arise in the future.

## Data availability statement

The original contributions presented in the study are included in the article/**Supplementary Material**. Further inquiries can be directed to the corresponding author.

## Ethics statement

The studies involving human participants were reviewed approved by a Central Ethics Committee, OLV Ziekenhuis Aalst, Belgium and the Ethics Committee of the Ghent University Hospital, Belgium. The patients/participants provided written informed consent to participate in this study.

## Author contributions

IL-R was the Principal Investigator of this study; CM, JJ, BJ, and GL-R were co-investigators of this study; GW was responsible for the humoral and cell-mediated immunoassays at CEVAC laboratory; VB was responsible for the humoral immunoassays at the laboratory of VRC. Concept and design: IL-R, GL-R, AD, FJ, VB, and FT; Acquisition, analysis, or interpretation of data: IL-R, CM, JJ, BJ, FJ, AD, YJ, GW, KT, VB, GL-R, and FT; Administrative, technical, or material support: IL-R, CM, JJ, BJ, FJ, AD, YJ, GW, SH, VB, GL-R, VK, HA, KH, FT; GL-R, and FT drafted the manuscript. All authors were involved in the drafting and review of the article, the decision to submit for publication and approved the submitted version.

## Acknowledgments

The authors are most grateful to the healthy volunteers who took part in this study. They especially acknowledge the contributions of the staff of the Center for Vaccinology (Ghent, Belgium). The authors are grateful for the contribution of the staff of the Vaccine Research Center (Tampere, Finland). The authors would like to thank the IQVIA CRO teams especially the Clinical Project Manager Nikoleta Stamboldzhieva. The authors would like to acknowledge Silke Kern for project management support. The authors are grateful for input to and discussion of the project with Timo Vesikari. The authors would also like to thank all other people involved.

## Conflict of interest

Author GL-R was retained as a consultant by Icon Genetics GmbH (Halle, Germany) to assist in the design and management of the trial, analysis of results and development of the manuscript. AD, FJ, FT, VK, HA, and KH are employees of Icon Genetics GmbH (Halle, Germany), a wholly owned subsidiary of DENKA Company, Ltd (Tokyo, Japan).

The remaining authors declare that the research was conducted in the absence of any commercial or financial relationships that could be construed as a potential conflict of interest.

The authors declare that this trial has received funding from DENKA Company, Ltd (Tokyo, Japan), through its wholly owned subsidiary company, Icon Genetics GmbH (Halle, Germany). The funder was involved in the study design, analysis and interpretation of data, the writing of this article, and the decision to submit it for publication.

## Publisher’s note

All claims expressed in this article are solely those of the authors and do not necessarily represent those of their affiliated organizations, or those of the publisher, the editors and the reviewers. Any product that may be evaluated in this article, or claim that may be made by its manufacturer, is not guaranteed or endorsed by the publisher.
