# Supplementary material for: A randomized, double-blind, placebo-controlled, dose-escalating phase I trial to evaluate safety and immunogenicity of a plant-produced, bivalent, recombinant norovirus-like particle vaccine
Source: Front Immunol. 2022 Oct 7;13:1021500. doi: 10.3389/fimmu.2022.1021500 (PMC9585308; doi:10.3389/fimmu.2022.1021500)
Supplement: Supplementary file 1 [file DataSheet_1.docx]

A randomized, dose-escalating Phase I trial to evaluate safety and immunogenicity of a Plant-produced, Bivalent, Recombinant Norovirus-like Particle Vaccine

**Isabel Leroux-Roels^1^, Cathy Maes^1^, Jasper Joye^1^, Bart Jacobs^1^, Franziska Jarczowski^2^, André Diessner^2^, Yorick Janssens^1^, Gwenn Waerlop^1^, Kirsi Tamminen^3^, Suvi Heinimäki^3^, Vesna Blazevic^3^, Geert Leroux-Roels^1^, Victor Klimyuk^2^, Hiroshi Adachi^2,4^, Kazuyuki Hiruta^2,4^, Frank Thieme^2*^**

^1^Center for Vaccinology (CEVAC), Ghent University and University Hospital, Ghent, Belgium.

^2^Icon Genetics GmbH, a Denka Company, Weinbergweg 22, D-06120 Halle, Germany

^3^Vaccine Research Center, University of Tampere, Arvo Ylpön katu 34, 33520 Tampere, Finland

^4^Denka Co., Ltd., Nihonbashi Mitsui Tower, 1-1, Nihonbashi-Muromachi 2-chome, Chuo-ku, Tokyo 103-8338, Japan

*** Correspondence:**Frank Thieme
thieme@icongenetics.de

Keywords: norovirus, virus-like particle, plant-produced, vaccine, clinical trial, adults, safety, immunogenicity.

**Supplementary Material**

[Supplementary Table 1: Inclusion and exclusion criteria 3](#_Toc109219825)

[Supplementary Table 2: Schedule of Assessments 5](#_Toc109219826)

[Supplementary Table 3: Incidence of Solicited Local Adverse Events Reported During the 7-day Post-vaccination Period Following Each Dose and Overall 8](#_Toc109219827)

[Supplementary Table 4: Incidence of Solicited General Adverse Events Reported During the 7-day Post-vaccination Period Following Each Dose and Overall 11](#_Toc109219828)

[Supplementary Table 5: Number and Percentage of Subjects Reporting the Occurrence of Unsolicited Adverse Events Classified by MedDRA Primary System Organ Class and Preferred Term During the 28-day Post-vaccination Period 19](#_Toc109219829)

[Supplementary Table 6: Stability data at +5 ± 3°C for rNV-2v clinical batches 22](#_Toc109219830)

[Supplementary Figure 1: Schematic of Study Design 23](#_Toc109211580)

[Supplementary Figure 2: Sentinel Dosing – rNV-2v (Cohort 1 and Cohort 2) 24](#_Toc109211581)

[Supplementary Figure 3: Evolutionary Relationships of Virus Protein 1 Protruding Domains amino acid sequence of Norovirus Virus-like Particles used in Enzyme-linked Immunosorbent Assays to Assess Cross-reactive Immunoglobulin G 25](#_Toc109211582)

**Supplementary Table 1:** Inclusion and exclusion criteria

| Inclusion Criteria |
| --- |
| 1. Capable of giving signed informed consent which included compliance with the requirements and restrictions listed in the Informed Consent Form and in the protocol. |
| 1. Male or female aged 18 to 40 years (inclusive) at Screening. |
| 1. Good general health as determined by a Screening evaluation that included vital signs, medical history, physical examinations, and laboratory assessments within 28 days before administration of the Investigational Medicinal Product (IMP). |
| 1. Expressed interest, availability, and understanding to fulfil the study requirements, and in the opinion of the Investigator, could and would comply with the protocol requirements. |
| 1. Female subjects of childbearing potential were to use a highly effective licensed method of birth control for 30 days prior to the first vaccination and had to agree to continue such precautions during the study until 60 days after the second vaccination. Male subjects had to agree to employ barrier contraception from the day of first vaccination until 60 days after the second vaccination, if their female partner of childbearing potential did not use a highly effective method of birth control. |
| 1. Agreed not to participate in another clinical study with an investigational product or to use any nonregistered product (drug, vaccine, or medical device) for the duration of the study (337 days after the last dose of study IMP). |
| Exclusion Criteria Any condition that could interfere with study participation or place the subject at increased risk of AE was excluded from the study. Additionally, subjects were excluded from the study if any of the following criteria applied: |
| 1. Positive serology test results for hepatitis C virus, human immunodeficiency virus antibody, and/or hepatitis B surface antigen at Screening. |
| 1. Pregnant or lactating women. |
| 1. History of any of the following medical illnesses, which, in the opinion of the Investigator, could interfere with study participation or place the subject at increased risk of AEs, including but not limited to: immunosuppression (disease or treatments that may affect immune system function), diabetes, cancer (malignancy other than a resolved or excised skin lesion), heart disease (hospitalization for a heart attack, arrhythmia, or syncope), unconsciousness (excluding single and brief concussion), seizures, asthma requiring treatment with inhaler or medication in the past 2 years, neuroinflammatory disease, autoimmune disease, recurrent infections (more than 3 hospitalizations for invasive bacterial infections such as pneumonia or meningitis). |
| 1. Any current or chronic conditions requiring daily medication other than vitamins, minerals, or antihypertensives, as per the Investigator’s discretion. Rescreening for acute illness that was expected to resolve quickly was allowed, as per the Investigator’s discretion. |
| 1. Known or suspected allergies or hypersensitivity to any component of the IMP. |
| 1. Any clinically significant abnormality detected during physical examinations or vital sign evaluations, as per the Investigator’s discretion. |
| 1. Hypertension defined as a mean of triplicate sitting blood pressure (BP) measurements >150/90 mm Hg.   Note: Subjects with chronic, stable, and well-controlled hypertension on medications were allowed. |
| 1. Any Screening hematology or biochemistry abnormalities considered clinically significant by the Investigator.   Note: Any abnormal laboratory evaluation could be repeated once (if out-of-range) at the Investigator’s discretion. If repeated and the values continued to be outside of the site’s normal ranges, could enroll if determined by the Investigator to be not clinically significant. |
| 1. For women of childbearing potential, positive urine or serum pregnancy test at Screening or within 24 hours of the first dose of the IMP.   Note: Serum pregnancy test was performed only at Screening. Urine pregnancy test was performed on the day of administration of each dose of the IMP. The IMP could only be administered if the pregnancy test was negative. Pregnancy test had to be performed even if the subject was menstruating at the time of the study visit. |
| Exclusion Criteria Any condition that could interfere with study participation or place the subject at increased risk of AE was excluded from the study. Additionally, subjects were excluded from the study if any of the following criteria applied: |
| 1. Body (oral) temperature >38°C or symptoms of an acute self-limited illness such as an upper respiratory infection or gastroenteritis within 3 days of administration of the first dose of the IMP. |
| 1. Administration of rNV-2v or any other experimental norovirus vaccine in the past. |
| 1. Planned administration of other vaccines from 14 days before the first administration of the IMP and 60 days after the last study administration of the IMP, with the exception of inactivated influenza vaccine, which could be administered up to 14 days before or from 30 days after the last administration of the IMP. |
| 1. Administration of immunoglobins or any blood products within 180 days prior to the first administration of the IMP and throughout the study duration, as per the Investigator’s discretion. |
| 1. Relatives of the Sponsor, clinical research organization, or the study center personnel were excluded from participating in the study. |
| 1. History of psychiatric hospitalization, alcohol abuse, or illicit drug use, which in the opinion of the Investigator, could affect the subject’s participation in the study. |
| 1. Other condition that in the clinical judgment of the Investigator would jeopardize the safety or rights of a subject participating in the study, would render the subject unable to comply with the protocol, or would interfere with the evaluation of the IMP. |

Supplementary Table 2: Schedule of Assessments

| Visit | 1 | 2 | Phone  Call^*^ | 3 | 4 | Phone Call* | 5 | 6 | 7 | 8 | Discontinuation Visit (if a subject discontinued before Day 365) |
| --- | --- | --- | --- | --- | --- | --- | --- | --- | --- | --- | --- |
| Week |  | | Week 1 | | Week 4 | Week 5 | | Week 8 | Week 26 | Week 52 |  |
| Day | −28 to −1 | 1 | 3 | 8 | 29 | 31 | 36 | 57 | 183 | 365 |  |
| Interval |  |  | Visit 1 + 2 (+2 days) | Day 1 + 7 (±1 day) | Day 1 + 28 (±3 days) | Day 29 +2 (+2 days) | Day 29 + 7 (±1 day) | Day 29 + 28 (±3 days) | Day 1 + 182 (±7 days) | Day 1 + 364 (±7 days) |  |
| Informed consent | X |  |  |  |  |  |  |  |  |  |  |
| Inclusion/exclusion criteria | X | X |  |  |  |  |  |  |  |  |  |
| Demographics^a^ | X |  |  |  |  |  |  |  |  |  |  |
| Medical and surgical history | X | X |  |  |  |  |  |  |  |  |  |
| Physical examination^b^ | X | X |  |  |  |  |  |  |  |  | X |
| Vital signs^c^ | X^d^ | X |  | X | X |  | X |  |  | X | X |
| Serum/urine pregnancy test for women of childbearing potential^e^ | X | X |  |  | X |  |  |  |  |  |  |
| Clinical Laboratory Tests^f^ | X^g^ | X |  | X | X |  | X | X |  |  |  |
| Serology^h^ | X |  |  |  |  |  |  |  |  |  |  |
| Randomisation |  | X |  |  |  |  |  |  |  |  |  |
| Vaccine (VLP)/placebo administration^i^ |  | X |  |  | X |  |  |  |  |  |  |
| Humoral immunogenicity^j^ |  | X |  | X | X |  |  | X | X | X |  |
| Cellular immunogenicity^k^ |  | X |  |  | X |  |  | X |  | X |  |
| Recording of solicited AEs^l^ |  | X |  | X | X |  | X |  |  |  | X |
| Recording unsolicited AEs^m^ |  | X |  | X | X |  | X | X |  |  |  |
| Diary cards |  | X |  | X | X |  | X |  |  |  |  |
| Recording of SAEs |  | X | X | X | X | X | X | X | X | X | X |
| Recording of prior and concomitant medication | X | X |  | X | X |  | X | X | X | X | X |
| Safety phone call |  |  | X |  |  | X |  |  |  |  |  |

AE = adverse event, BP = blood pressure, Ig = immunoglobin, IMP = investigational medicinal product, SAE = serious adverse event, VLP = virus-like particle.

* Only applicable to the sentinel group and performed at least 48 hours after each IMP administration in both cohorts.

1. Age, sex, and race were recorded.
2. Physical examinations were performed, and any clinically significant changes/findings were recorded as AEs after first dose of the IMP and recorded as medical history prior to first IMP administration. Physical examinations included the examination of the following: general appearance, weight and height, abdomen; head and neck; eyes, ears, nose, throat, chest/respiratory, heart/cardiovascular, gastrointestinal/liver, musculoskeletal/extremities, dermatological/skin, thyroid, lymph nodes, and neurological (at Screening). On Day 1, a brief physical examination was performed only if there were any symptoms that required further evaluation.
3. Vital signs (including BP [systolic and diastolic], pulse rate, and body temperature [oral]) were measured in a sitting position after the subject had rested comfortably for at least 5 minutes using an automated instrument. Additional vital signs measurements could be performed for the safety of the subjects. On the days of dosing, these were performed prior to dosing. On all other days, these were performed when the subject returned to the center.
4. Triplicate BP measurements were performed at Screening with subjects in a sitting position, a mean value of the measurement was considered for eligibility assessments.
5. For female subjects of childbearing potential, a serum pregnancy test was performed only at Screening. Urine pregnancy test was performed on the day of administration of each dose of the IMP. The IMP could only be administered if the pregnancy test was negative. Pregnancy test had to be performed even if the subject was menstruating at the time of the study visit.
6. Clinical laboratory tests included hematology and serum chemistry. The following laboratory evaluations had to be evaluated at Screening: white blood cell count, neutrophil count, platelets, hemoglobin, alanine aminotransferase, aspartate aminotransferase, gamma glutamyl transferase, alkaline phosphatase, bilirubin (total and indirect), and creatinine. On the days of dosing, these were performed prior to dosing.
7. Urine analysis (red blood cells, protein, glucose) was performed.
8. Serology tests for the hepatitis C virus, human immunodeficiency virus antibody, and/or hepatitis B surface antigen were performed.
9. After each dosing, all subjects were kept in observation for at least 1 hour, with appropriate treatment readily available in case of anaphylaxis.
10. Samples for humoral immunogenicity were collected pre-dose, and on Week 1, Week 4, Week 8, Week 26, and Week 52 visits.
11. Samples for cellular immunogenicity were collected pre-dose, and on Week 4, Week 8, and Week 52 visits.
12. Solicited AEs including injection site pain, redness, and swelling and oral temperature were captured in the diary card for 7 days (ie, Day 1 to Day 7 and Day 29 to Day 36, inclusive) after each IMP administration. Subjects were trained in the use of the diary card. General solicited AEs included fever, fatigue, gastrointestinal symptoms (nausea, vomiting, diarrhoea, and/or abdominal pain), headache, myalgia, shivering, and arthralgia.
13. Unsolicited AEs were recorded up to 1 month after each IMP administration.

| **Supplementary Table 3:** Incidence of Solicited Local Adverse Events Reported During the 7-day Post-vaccination Period Following Each Dose and Overall | | | | | | | | | | | | | | | | | | |
| --- | --- | --- | --- | --- | --- | --- | --- | --- | --- | --- | --- | --- | --- | --- | --- | --- | --- | --- |
|  | | rNV-2v [50 µg GI.4 + 50 µg GII.4] | | | | |  | rNV-2v [150 µg GI.4 + 150 µg GII.4] | | | | |  | Placebo | | | | |
| Symptom | Type | N | n | % | 95%  LL | 95%  UL |  | N | n | % | 95%  LL | 95%  UL |  | N | n | % | 95%  LL | 95%  UL |
|  |  |  |  |  |  |  |  |  |  |  |  |  |  |  |  |  |  |  |
| Dose 1 |  |  |  |  |  |  |  |  |  |  |  |  |  |  |  |  |  |  |
| Pain | All | 20 | 5 | 25.0 | 8.7 | 49.1 |  | 20 | 3 | 15.0 | 3.2 | 37.9 |  | 20 | 2 | 10.0 | 1.2 | 31.7 |
|  | Severe | 20 | 0 | 0 | 0.0 | 16.8 |  | 20 | 0 | 0 | 0.0 | 16.8 |  | 20 | 0 | 0 | 0.0 | 16.8 |
|  | Medically Attended Visit | 20 | 0 | 0 | 0.0 | 16.8 |  | 20 | 0 | 0 | 0.0 | 16.8 |  | 20 | 0 | 0 | 0.0 | 16.8 |
|  |  |  |  |  |  |  |  |  |  |  |  |  |  |  |  |  |  |  |
| Redness | All | 20 | 1 | 5.0 | 0.1 | 24.9 |  | 20 | 0 | 0 | 0.0 | 16.8 |  | 20 | 2 | 10.0 | 1.2 | 31.7 |
|  | Severe | 20 | 0 | 0 | 0.0 | 16.8 |  | 20 | 0 | 0 | 0.0 | 16.8 |  | 20 | 0 | 0 | 0.0 | 16.8 |
|  | Medically Attended Visit | 20 | 0 | 0 | 0.0 | 16.8 |  | 20 | 0 | 0 | 0.0 | 16.8 |  | 20 | 0 | 0 | 0.0 | 16.8 |
|  |  |  |  |  |  |  |  |  |  |  |  |  |  |  |  |  |  |  |
| Swelling | All | 20 | 0 | 0 | 0.0 | 16.8 |  | 20 | 0 | 0 | 0.0 | 16.8 |  | 20 | 0 | 0 | 0.0 | 16.8 |
|  | Severe | 20 | 0 | 0 | 0.0 | 16.8 |  | 20 | 0 | 0 | 0.0 | 16.8 |  | 20 | 0 | 0 | 0.0 | 16.8 |
|  | Medically Attended Visit | 20 | 0 | 0 | 0.0 | 16.8 |  | 20 | 0 | 0 | 0.0 | 16.8 |  | 20 | 0 | 0 | 0.0 | 16.8 |
|  |  |  |  |  |  |  |  |  |  |  |  |  |  |  |  |  |  |  |
| Dose 2 |  |  |  |  |  |  |  |  |  |  |  |  |  |  |  |  |  |  |
| Pain | All | 20 | 8 | 40.0 | 19.1 | 63.9 |  | 20 | 12 | 60.0 | 36.1 | 80.9 |  | 20 | 1 | 5.0 | 0.1 | 24.9 |
|  | Severe | 20 | 0 | 0 | 0.0 | 16.8 |  | 20 | 0 | 0 | 0.0 | 16.8 |  | 20 | 0 | 0 | 0.0 | 16.8 |
|  | Medically Attended Visit | 20 | 0 | 0 | 0.0 | 16.8 |  | 20 | 0 | 0 | 0.0 | 16.8 |  | 20 | 0 | 0 | 0.0 | 16.8 |
|  |  |  |  |  |  |  |  |  |  |  |  |  |  |  |  |  |  |  |
| Note(s): For each dose and overall/subject: N = number of subjects with at least one documented dose; n/% = number/percentage of subjects reporting at least once the symptom. For Overall/dose: N = number of documented doses; n/% = number/percentage of doses followed by at least one type of symptom. 95% CI = exact 95% confidence interval; LL = lower limit; UL = upper limit. | | | | | | | | | | | | | | | | | | |

|  | | rNV-2v [50 µg GI.4 + 50 µg GII.4] | | | | |  | rNV-2v [150 µg GI.4 + 150 µg GII.4] | | | | |  | Placebo | | | | |
| --- | --- | --- | --- | --- | --- | --- | --- | --- | --- | --- | --- | --- | --- | --- | --- | --- | --- | --- |
| Symptom | Type | N | n | % | 95%  LL | 95%  UL |  | N | n | % | 95%  LL | 95%  UL |  | N | n | % | 95%  LL | 95%  UL |
|  |  |  |  |  |  |  |  |  |  |  |  |  |  |  |  |  |  |  |
| Dose 2 |  |  |  |  |  |  |  |  |  |  |  |  |  |  |  |  |  |  |
| Redness | All | 20 | 0 | 0 | 0.0 | 16.8 |  | 20 | 0 | 0 | 0.0 | 16.8 |  | 20 | 1 | 5.0 | 0.1 | 24.9 |
|  | Severe | 20 | 0 | 0 | 0.0 | 16.8 |  | 20 | 0 | 0 | 0.0 | 16.8 |  | 20 | 0 | 0 | 0.0 | 16.8 |
|  | Medically Attended Visit | 20 | 0 | 0 | 0.0 | 16.8 |  | 20 | 0 | 0 | 0.0 | 16.8 |  | 20 | 0 | 0 | 0.0 | 16.8 |
|  |  |  |  |  |  |  |  |  |  |  |  |  |  |  |  |  |  |  |
| Swelling | All | 20 | 0 | 0 | 0.0 | 16.8 |  | 20 | 0 | 0 | 0.0 | 16.8 |  | 20 | 1 | 5.0 | 0.1 | 24.9 |
|  | Severe | 20 | 0 | 0 | 0.0 | 16.8 |  | 20 | 0 | 0 | 0.0 | 16.8 |  | 20 | 0 | 0 | 0.0 | 16.8 |
|  | Medically Attended Visit | 20 | 0 | 0 | 0.0 | 16.8 |  | 20 | 0 | 0 | 0.0 | 16.8 |  | 20 | 0 | 0 | 0.0 | 16.8 |
|  |  |  |  |  |  |  |  |  |  |  |  |  |  |  |  |  |  |  |
| Overall/Dose |  |  |  |  |  |  |  |  |  |  |  |  |  |  |  |  |  |  |
| Pain | All | 40 | 13 | 32.5 | 18.6 | 49.1 |  | 40 | 15 | 37.5 | 22.7 | 54.2 |  | 40 | 3 | 7.5 | 1.6 | 20.4 |
|  | Severe | 40 | 0 | 0 | 0.0 | 8.8 |  | 40 | 0 | 0 | 0.0 | 8.8 |  | 40 | 0 | 0 | 0.0 | 8.8 |
|  | Medically Attended Visit | 40 | 0 | 0 | 0.0 | 8.8 |  | 40 | 0 | 0 | 0.0 | 8.8 |  | 40 | 0 | 0 | 0.0 | 8.8 |
|  |  |  |  |  |  |  |  |  |  |  |  |  |  |  |  |  |  |  |
| Redness | All | 40 | 1 | 2.5 | 0.1 | 13.2 |  | 40 | 0 | 0 | 0.0 | 8.8 |  | 40 | 3 | 7.5 | 1.6 | 20.4 |
|  | Severe | 40 | 0 | 0 | 0.0 | 8.8 |  | 40 | 0 | 0 | 0.0 | 8.8 |  | 40 | 0 | 0 | 0.0 | 8.8 |
|  | Medically Attended Visit | 40 | 0 | 0 | 0.0 | 8.8 |  | 40 | 0 | 0 | 0.0 | 8.8 |  | 40 | 0 | 0 | 0.0 | 8.8 |
|  |  |  |  |  |  |  |  |  |  |  |  |  |  |  |  |  |  |  |
| Note(s): For each dose and overall/subject: N = number of subjects with at least one documented dose; n/% = number/percentage of subjects reporting at least once the symptom. For Overall/dose: N = number of documented doses; n/% = number/percentage of doses followed by at least one type of symptom. 95% CI = exact 95% confidence interval; LL = lower limit; UL = upper limit. | | | | | | | | | | | | | | | | | | |

|  | | rNV-2v [50 µg GI.4 + 50 µg GII.4] | | | | |  | rNV-2v [150 µg GI.4 + 150 µg GII.4] | | | | |  | Placebo | | | | |
| --- | --- | --- | --- | --- | --- | --- | --- | --- | --- | --- | --- | --- | --- | --- | --- | --- | --- | --- |
| Symptom | Type | N | n | % | 95%  LL | 95%  UL |  | N | n | % | 95%  LL | 95%  UL |  | N | n | % | 95%  LL | 95%  UL |
|  |  |  |  |  |  |  |  |  |  |  |  |  |  |  |  |  |  |  |
| Overall/Dose |  |  |  |  |  |  |  |  |  |  |  |  |  |  |  |  |  |  |
| Swelling | All | 40 | 0 | 0 | 0.0 | 8.8 |  | 40 | 0 | 0 | 0.0 | 8.8 |  | 40 | 1 | 2.5 | 0.1 | 13.2 |
|  | Severe | 40 | 0 | 0 | 0.0 | 8.8 |  | 40 | 0 | 0 | 0.0 | 8.8 |  | 40 | 0 | 0 | 0.0 | 8.8 |
|  | Medically Attended Visit | 40 | 0 | 0 | 0.0 | 8.8 |  | 40 | 0 | 0 | 0.0 | 8.8 |  | 40 | 0 | 0 | 0.0 | 8.8 |
|  |  |  |  |  |  |  |  |  |  |  |  |  |  |  |  |  |  |  |
| Overall/Subject |  |  |  |  |  |  |  |  |  |  |  |  |  |  |  |  |  |  |
| Pain | All | 20 | 10 | 50.0 | 27.2 | 72.8 |  | 20 | 12 | 60.0 | 36.1 | 80.9 |  | 20 | 2 | 10.0 | 1.2 | 31.7 |
|  | Severe | 20 | 0 | 0 | 0.0 | 16.8 |  | 20 | 0 | 0 | 0.0 | 16.8 |  | 20 | 0 | 0 | 0.0 | 16.8 |
|  | Medically Attended Visit | 20 | 0 | 0 | 0.0 | 16.8 |  | 20 | 0 | 0 | 0.0 | 16.8 |  | 20 | 0 | 0 | 0.0 | 16.8 |
|  |  |  |  |  |  |  |  |  |  |  |  |  |  |  |  |  |  |  |
| Redness | All | 20 | 1 | 5.0 | 0.1 | 24.9 |  | 20 | 0 | 0 | 0.0 | 16.8 |  | 20 | 3 | 15.0 | 3.2 | 37.9 |
|  | Severe | 20 | 0 | 0 | 0.0 | 16.8 |  | 20 | 0 | 0 | 0.0 | 16.8 |  | 20 | 0 | 0 | 0.0 | 16.8 |
|  | Medically Attended Visit | 20 | 0 | 0 | 0.0 | 16.8 |  | 20 | 0 | 0 | 0.0 | 16.8 |  | 20 | 0 | 0 | 0.0 | 16.8 |
|  |  |  |  |  |  |  |  |  |  |  |  |  |  |  |  |  |  |  |
| Swelling | All | 20 | 0 | 0 | 0.0 | 16.8 |  | 20 | 0 | 0 | 0.0 | 16.8 |  | 20 | 1 | 5.0 | 0.1 | 24.9 |
|  | Severe | 20 | 0 | 0 | 0.0 | 16.8 |  | 20 | 0 | 0 | 0.0 | 16.8 |  | 20 | 0 | 0 | 0.0 | 16.8 |
|  | Medically Attended Visit | 20 | 0 | 0 | 0.0 | 16.8 |  | 20 | 0 | 0 | 0.0 | 16.8 |  | 20 | 0 | 0 | 0.0 | 16.8 |
|  |  |  |  |  |  |  |  |  |  |  |  |  |  |  |  |  |  |  |
| Note(s): For each dose and overall/subject: N = number of subjects with at least one documented dose; n/% = number/percentage of subjects reporting at least once the symptom. For Overall/dose: N = number of documented doses; n/% = number/percentage of doses followed by at least one type of symptom. 95% CI = exact 95% confidence interval; LL = lower limit; UL = upper limit. | | | | | | | | | | | | | | | | | | |

| **Supplementary Table 4:** Incidence of Solicited General Adverse Events Reported During the 7-day Post-vaccination Period Following Each Dose and Overall | | | | | | | | | | | | | | | | | | |
| --- | --- | --- | --- | --- | --- | --- | --- | --- | --- | --- | --- | --- | --- | --- | --- | --- | --- | --- |
|  | | rNV-2v [50 µg GI.4 + 50 µg GII.4] | | | | |  | rNV-2v [150 µg GI.4 + 150 µg GII.4] | | | | |  | Placebo | | | | |
| Symptom | Type | N | n | % | 95%  LL | 95%  UL |  | N | n | % | 95%  LL | 95%  UL |  | N | n | % | 95%  LL | 95%  UL |
|  |  |  |  |  |  |  |  |  |  |  |  |  |  |  |  |  |  |  |
| Dose 1 |  |  |  |  |  |  |  |  |  |  |  |  |  |  |  |  |  |  |
| Arthralgia | All | 20 | 0 | 0 | 0.0 | 16.8 |  | 20 | 1 | 5.0 | 0.1 | 24.9 |  | 20 | 0 | 0 | 0.0 | 16.8 |
|  | Severe | 20 | 0 | 0 | 0.0 | 16.8 |  | 20 | 0 | 0 | 0.0 | 16.8 |  | 20 | 0 | 0 | 0.0 | 16.8 |
|  | Related | 20 | 0 | 0 | 0.0 | 16.8 |  | 20 | 0 | 0 | 0.0 | 16.8 |  | 20 | 0 | 0 | 0.0 | 16.8 |
|  | Severe*Related | 20 | 0 | 0 | 0.0 | 16.8 |  | 20 | 0 | 0 | 0.0 | 16.8 |  | 20 | 0 | 0 | 0.0 | 16.8 |
|  | Medically Attended Visit | 20 | 0 | 0 | 0.0 | 16.8 |  | 20 | 0 | 0 | 0.0 | 16.8 |  | 20 | 0 | 0 | 0.0 | 16.8 |
|  |  |  |  |  |  |  |  |  |  |  |  |  |  |  |  |  |  |  |
| Fatigue | All | 20 | 5 | 25.0 | 8.7 | 49.1 |  | 20 | 5 | 25.0 | 8.7 | 49.1 |  | 20 | 6 | 30.0 | 11.9 | 54.3 |
|  | Severe | 20 | 1 | 5.0 | 0.1 | 24.9 |  | 20 | 0 | 0 | 0.0 | 16.8 |  | 20 | 1 | 5.0 | 0.1 | 24.9 |
|  | Related | 20 | 3 | 15.0 | 3.2 | 37.9 |  | 20 | 4 | 20.0 | 5.7 | 43.7 |  | 20 | 4 | 20.0 | 5.7 | 43.7 |
|  | Severe*Related | 20 | 1 | 5.0 | 0.1 | 24.9 |  | 20 | 0 | 0 | 0.0 | 16.8 |  | 20 | 0 | 0 | 0.0 | 16.8 |
|  | Medically Attended Visit | 20 | 0 | 0 | 0.0 | 16.8 |  | 20 | 0 | 0 | 0.0 | 16.8 |  | 20 | 0 | 0 | 0.0 | 16.8 |
|  |  |  |  |  |  |  |  |  |  |  |  |  |  |  |  |  |  |  |
| Gastrointestinal Symptoms | All | 20 | 3 | 15.0 | 3.2 | 37.9 |  | 20 | 4 | 20.0 | 5.7 | 43.7 |  | 20 | 5 | 25.0 | 8.7 | 49.1 |
|  | Severe | 20 | 0 | 0 | 0.0 | 16.8 |  | 20 | 0 | 0 | 0.0 | 16.8 |  | 20 | 1 | 5.0 | 0.1 | 24.9 |
|  | Related | 20 | 2 | 10.0 | 1.2 | 31.7 |  | 20 | 4 | 20.0 | 5.7 | 43.7 |  | 20 | 3 | 15.0 | 3.2 | 37.9 |
|  | Severe*Related | 20 | 0 | 0 | 0.0 | 16.8 |  | 20 | 0 | 0 | 0.0 | 16.8 |  | 20 | 0 | 0 | 0.0 | 16.8 |
|  | Medically Attended Visit | 20 | 0 | 0 | 0.0 | 16.8 |  | 20 | 1 | 5.0 | 0.1 | 24.9 |  | 20 | 0 | 0 | 0.0 | 16.8 |
|  |  |  |  |  |  |  |  |  |  |  |  |  |  |  |  |  |  |  |
| Note(s): For each dose and overall/subject: N = number of subjects with at least one documented dose; n/% = number/percentage of subjects reporting at least once the symptom. For Overall/dose: N = number of documented doses; n/% = number/percentage of doses followed by at least one type of symptom. 95% CI = exact 95% confidence interval; LL = lower limit; UL = upper limit. | | | | | | | | | | | | | | | | | | |

|  | | rNV-2v [50 µg GI.4 + 50 µg GII.4] | | | | |  | rNV-2v [150 µg GI.4 + 150 µg GII.4] | | | | |  | Placebo | | | | |
| --- | --- | --- | --- | --- | --- | --- | --- | --- | --- | --- | --- | --- | --- | --- | --- | --- | --- | --- |
| Symptom | Type | N | n | % | 95%  LL | 95%  UL |  | N | n | % | 95%  LL | 95%  UL |  | N | n | % | 95%  LL | 95%  UL |
|  |  |  |  |  |  |  |  |  |  |  |  |  |  |  |  |  |  |  |
| Dose 1 |  |  |  |  |  |  |  |  |  |  |  |  |  |  |  |  |  |  |
| Headache | All | 20 | 4 | 20.0 | 5.7 | 43.7 |  | 20 | 7 | 35.0 | 15.4 | 59.2 |  | 20 | 7 | 35.0 | 15.4 | 59.2 |
|  | Severe | 20 | 1 | 5.0 | 0.1 | 24.9 |  | 20 | 0 | 0 | 0.0 | 16.8 |  | 20 | 1 | 5.0 | 0.1 | 24.9 |
|  | Related | 20 | 3 | 15.0 | 3.2 | 37.9 |  | 20 | 5 | 25.0 | 8.7 | 49.1 |  | 20 | 4 | 20.0 | 5.7 | 43.7 |
|  | Severe*Related | 20 | 1 | 5.0 | 0.1 | 24.9 |  | 20 | 0 | 0 | 0.0 | 16.8 |  | 20 | 0 | 0 | 0.0 | 16.8 |
|  | Medically Attended Visit | 20 | 0 | 0 | 0.0 | 16.8 |  | 20 | 0 | 0 | 0.0 | 16.8 |  | 20 | 0 | 0 | 0.0 | 16.8 |
|  |  |  |  |  |  |  |  |  |  |  |  |  |  |  |  |  |  |  |
| Myalgia | All | 20 | 2 | 10.0 | 1.2 | 31.7 |  | 20 | 1 | 5.0 | 0.1 | 24.9 |  | 20 | 0 | 0 | 0.0 | 16.8 |
|  | Severe | 20 | 0 | 0 | 0.0 | 16.8 |  | 20 | 0 | 0 | 0.0 | 16.8 |  | 20 | 0 | 0 | 0.0 | 16.8 |
|  | Related | 20 | 1 | 5.0 | 0.1 | 24.9 |  | 20 | 0 | 0 | 0.0 | 16.8 |  | 20 | 0 | 0 | 0.0 | 16.8 |
|  | Severe*Related | 20 | 0 | 0 | 0.0 | 16.8 |  | 20 | 0 | 0 | 0.0 | 16.8 |  | 20 | 0 | 0 | 0.0 | 16.8 |
|  | Medically Attended Visit | 20 | 0 | 0 | 0.0 | 16.8 |  | 20 | 0 | 0 | 0.0 | 16.8 |  | 20 | 0 | 0 | 0.0 | 16.8 |
|  |  |  |  |  |  |  |  |  |  |  |  |  |  |  |  |  |  |  |
| Shivering | All | 20 | 2 | 10.0 | 1.2 | 31.7 |  | 20 | 0 | 0 | 0.0 | 16.8 |  | 20 | 1 | 5.0 | 0.1 | 24.9 |
|  | Severe | 20 | 0 | 0 | 0.0 | 16.8 |  | 20 | 0 | 0 | 0.0 | 16.8 |  | 20 | 0 | 0 | 0.0 | 16.8 |
|  | Related | 20 | 1 | 5.0 | 0.1 | 24.9 |  | 20 | 0 | 0 | 0.0 | 16.8 |  | 20 | 0 | 0 | 0.0 | 16.8 |
|  | Severe*Related | 20 | 0 | 0 | 0.0 | 16.8 |  | 20 | 0 | 0 | 0.0 | 16.8 |  | 20 | 0 | 0 | 0.0 | 16.8 |
|  | Medically Attended Visit | 20 | 0 | 0 | 0.0 | 16.8 |  | 20 | 0 | 0 | 0.0 | 16.8 |  | 20 | 0 | 0 | 0.0 | 16.8 |
|  |  |  |  |  |  |  |  |  |  |  |  |  |  |  |  |  |  |  |
| Temperature | All | 20 | 0 | 0 | 0.0 | 16.8 |  | 20 | 0 | 0 | 0.0 | 16.8 |  | 20 | 0 | 0 | 0.0 | 16.8 |
|  |  |  |  |  |  |  |  |  |  |  |  |  |  |  |  |  |  |  |
| Note(s): For each dose and overall/subject: N = number of subjects with at least one documented dose; n/% = number/percentage of subjects reporting at least once the symptom. For Overall/dose: N = number of documented doses; n/% = number/percentage of doses followed by at least one type of symptom. 95% CI = exact 95% confidence interval; LL = lower limit; UL = upper limit. | | | | | | | | | | | | | | | | | | |

|  | | rNV-2v [50 µg GI.4 + 50 µg GII.4] | | | | |  | rNV-2v [150 µg GI.4 + 150 µg GII.4] | | | | |  | Placebo | | | | |
| --- | --- | --- | --- | --- | --- | --- | --- | --- | --- | --- | --- | --- | --- | --- | --- | --- | --- | --- |
| Symptom | Type | N | n | % | 95%  LL | 95%  UL |  | N | n | % | 95%  LL | 95%  UL |  | N | n | % | 95%  LL | 95%  UL |
|  |  |  |  |  |  |  |  |  |  |  |  |  |  |  |  |  |  |  |
| Dose 2 |  |  |  |  |  |  |  |  |  |  |  |  |  |  |  |  |  |  |
| Arthralgia | All | 20 | 0 | 0 | 0.0 | 16.8 |  | 20 | 0 | 0 | 0.0 | 16.8 |  | 20 | 0 | 0 | 0.0 | 16.8 |
|  | Severe | 20 | 0 | 0 | 0.0 | 16.8 |  | 20 | 0 | 0 | 0.0 | 16.8 |  | 20 | 0 | 0 | 0.0 | 16.8 |
|  | Related | 20 | 0 | 0 | 0.0 | 16.8 |  | 20 | 0 | 0 | 0.0 | 16.8 |  | 20 | 0 | 0 | 0.0 | 16.8 |
|  | Severe*Related | 20 | 0 | 0 | 0.0 | 16.8 |  | 20 | 0 | 0 | 0.0 | 16.8 |  | 20 | 0 | 0 | 0.0 | 16.8 |
|  | Medically Attended Visit | 20 | 0 | 0 | 0.0 | 16.8 |  | 20 | 0 | 0 | 0.0 | 16.8 |  | 20 | 0 | 0 | 0.0 | 16.8 |
|  |  |  |  |  |  |  |  |  |  |  |  |  |  |  |  |  |  |  |
| Fatigue | All | 20 | 3 | 15.0 | 3.2 | 37.9 |  | 20 | 2 | 10.0 | 1.2 | 31.7 |  | 20 | 8 | 40.0 | 19.1 | 63.9 |
|  | Severe | 20 | 0 | 0 | 0.0 | 16.8 |  | 20 | 0 | 0 | 0.0 | 16.8 |  | 20 | 0 | 0 | 0.0 | 16.8 |
|  | Related | 20 | 3 | 15.0 | 3.2 | 37.9 |  | 20 | 1 | 5.0 | 0.1 | 24.9 |  | 20 | 7 | 35.0 | 15.4 | 59.2 |
|  | Severe*Related | 20 | 0 | 0 | 0.0 | 16.8 |  | 20 | 0 | 0 | 0.0 | 16.8 |  | 20 | 0 | 0 | 0.0 | 16.8 |
|  | Medically Attended Visit | 20 | 0 | 0 | 0.0 | 16.8 |  | 20 | 0 | 0 | 0.0 | 16.8 |  | 20 | 0 | 0 | 0.0 | 16.8 |
|  |  |  |  |  |  |  |  |  |  |  |  |  |  |  |  |  |  |  |
| Gastrointestinal Symptoms | All | 20 | 1 | 5.0 | 0.1 | 24.9 |  | 20 | 2 | 10.0 | 1.2 | 31.7 |  | 20 | 4 | 20.0 | 5.7 | 43.7 |
|  | Severe | 20 | 0 | 0 | 0.0 | 16.8 |  | 20 | 0 | 0 | 0.0 | 16.8 |  | 20 | 0 | 0 | 0.0 | 16.8 |
|  | Related | 20 | 0 | 0 | 0.0 | 16.8 |  | 20 | 2 | 10.0 | 1.2 | 31.7 |  | 20 | 3 | 15.0 | 3.2 | 37.9 |
|  | Severe*Related | 20 | 0 | 0 | 0.0 | 16.8 |  | 20 | 0 | 0 | 0.0 | 16.8 |  | 20 | 0 | 0 | 0.0 | 16.8 |
|  | Medically Attended Visit | 20 | 1 | 5.0 | 0.1 | 24.9 |  | 20 | 0 | 0 | 0.0 | 16.8 |  | 20 | 0 | 0 | 0.0 | 16.8 |
|  |  |  |  |  |  |  |  |  |  |  |  |  |  |  |  |  |  |  |
| Note(s): For each dose and overall/subject: N = number of subjects with at least one documented dose; n/% = number/percentage of subjects reporting at least once the symptom. For Overall/dose: N = number of documented doses; n/% = number/percentage of doses followed by at least one type of symptom. 95% CI = exact 95% confidence interval; LL = lower limit; UL = upper limit. | | | | | | | | | | | | | | | | | | |

|  | | rNV-2v [50 µg GI.4 + 50 µg GII.4] | | | | |  | rNV-2v [150 µg GI.4 + 150 µg GII.4] | | | | |  | Placebo | | | | |
| --- | --- | --- | --- | --- | --- | --- | --- | --- | --- | --- | --- | --- | --- | --- | --- | --- | --- | --- |
| Symptom | Type | N | n | % | 95%  LL | 95%  UL |  | N | n | % | 95%  LL | 95%  UL |  | N | n | % | 95%  LL | 95%  UL |
|  |  |  |  |  |  |  |  |  |  |  |  |  |  |  |  |  |  |  |
| Dose 2 |  |  |  |  |  |  |  |  |  |  |  |  |  |  |  |  |  |  |
| Headache | All | 20 | 4 | 20.0 | 5.7 | 43.7 |  | 20 | 1 | 5.0 | 0.1 | 24.9 |  | 20 | 6 | 30.0 | 11.9 | 54.3 |
|  | Severe | 20 | 0 | 0 | 0.0 | 16.8 |  | 20 | 0 | 0 | 0.0 | 16.8 |  | 20 | 0 | 0 | 0.0 | 16.8 |
|  | Related | 20 | 2 | 10.0 | 1.2 | 31.7 |  | 20 | 1 | 5.0 | 0.1 | 24.9 |  | 20 | 5 | 25.0 | 8.7 | 49.1 |
|  | Severe*Related | 20 | 0 | 0 | 0.0 | 16.8 |  | 20 | 0 | 0 | 0.0 | 16.8 |  | 20 | 0 | 0 | 0.0 | 16.8 |
|  | Medically Attended Visit | 20 | 1 | 5.0 | 0.1 | 24.9 |  | 20 | 0 | 0 | 0.0 | 16.8 |  | 20 | 3 | 15.0 | 3.2 | 37.9 |
|  |  |  |  |  |  |  |  |  |  |  |  |  |  |  |  |  |  |  |
| Myalgia | All | 20 | 1 | 5.0 | 0.1 | 24.9 |  | 20 | 0 | 0 | 0.0 | 16.8 |  | 20 | 3 | 15.0 | 3.2 | 37.9 |
|  | Severe | 20 | 0 | 0 | 0.0 | 16.8 |  | 20 | 0 | 0 | 0.0 | 16.8 |  | 20 | 0 | 0 | 0.0 | 16.8 |
|  | Related | 20 | 0 | 0 | 0.0 | 16.8 |  | 20 | 0 | 0 | 0.0 | 16.8 |  | 20 | 3 | 15.0 | 3.2 | 37.9 |
|  | Severe*Related | 20 | 0 | 0 | 0.0 | 16.8 |  | 20 | 0 | 0 | 0.0 | 16.8 |  | 20 | 0 | 0 | 0.0 | 16.8 |
|  | Medically Attended Visit | 20 | 1 | 5.0 | 0.1 | 24.9 |  | 20 | 0 | 0 | 0.0 | 16.8 |  | 20 | 0 | 0 | 0.0 | 16.8 |
|  |  |  |  |  |  |  |  |  |  |  |  |  |  |  |  |  |  |  |
| Shivering | All | 20 | 0 | 0 | 0.0 | 16.8 |  | 20 | 0 | 0 | 0.0 | 16.8 |  | 20 | 1 | 5.0 | 0.1 | 24.9 |
|  | Severe | 20 | 0 | 0 | 0.0 | 16.8 |  | 20 | 0 | 0 | 0.0 | 16.8 |  | 20 | 0 | 0 | 0.0 | 16.8 |
|  | Related | 20 | 0 | 0 | 0.0 | 16.8 |  | 20 | 0 | 0 | 0.0 | 16.8 |  | 20 | 1 | 5.0 | 0.1 | 24.9 |
|  | Severe*Related | 20 | 0 | 0 | 0.0 | 16.8 |  | 20 | 0 | 0 | 0.0 | 16.8 |  | 20 | 0 | 0 | 0.0 | 16.8 |
|  | Medically Attended Visit | 20 | 0 | 0 | 0.0 | 16.8 |  | 20 | 0 | 0 | 0.0 | 16.8 |  | 20 | 0 | 0 | 0.0 | 16.8 |
|  |  |  |  |  |  |  |  |  |  |  |  |  |  |  |  |  |  |  |
| Temperature | All | 20 | 0 | 0 | 0.0 | 16.8 |  | 20 | 0 | 0 | 0.0 | 16.8 |  | 20 | 0 | 0 | 0.0 | 16.8 |
|  |  |  |  |  |  |  |  |  |  |  |  |  |  |  |  |  |  |  |
| Note(s): For each dose and overall/subject: N = number of subjects with at least one documented dose; n/% = number/percentage of subjects reporting at least once the symptom. For Overall/dose: N = number of documented doses; n/% = number/percentage of doses followed by at least one type of symptom. 95% CI = exact 95% confidence interval; LL = lower limit; UL = upper limit. | | | | | | | | | | | | | | | | | | |

|  | | rNV-2v [50 µg GI.4 + 50 µg GII.4] | | | | |  | rNV-2v [150 µg GI.4 + 150 µg GII.4] | | | | |  | Placebo | | | | |
| --- | --- | --- | --- | --- | --- | --- | --- | --- | --- | --- | --- | --- | --- | --- | --- | --- | --- | --- |
| Symptom | Type | N | n | % | 95%  LL | 95%  UL |  | N | n | % | 95%  LL | 95%  UL |  | N | n | % | 95%  LL | 95%  UL |
|  |  |  |  |  |  |  |  |  |  |  |  |  |  |  |  |  |  |  |
| Overall/Dose |  |  |  |  |  |  |  |  |  |  |  |  |  |  |  |  |  |  |
| Arthralgia | All | 40 | 0 | 0 | 0.0 | 8.8 |  | 40 | 1 | 2.5 | 0.1 | 13.2 |  | 40 | 0 | 0 | 0.0 | 8.8 |
|  | Severe | 40 | 0 | 0 | 0.0 | 8.8 |  | 40 | 0 | 0 | 0.0 | 8.8 |  | 40 | 0 | 0 | 0.0 | 8.8 |
|  | Related | 40 | 0 | 0 | 0.0 | 8.8 |  | 40 | 0 | 0 | 0.0 | 8.8 |  | 40 | 0 | 0 | 0.0 | 8.8 |
|  | Severe*Related | 40 | 0 | 0 | 0.0 | 8.8 |  | 40 | 0 | 0 | 0.0 | 8.8 |  | 40 | 0 | 0 | 0.0 | 8.8 |
|  | Medically Attended Visit | 40 | 0 | 0 | 0.0 | 8.8 |  | 40 | 0 | 0 | 0.0 | 8.8 |  | 40 | 0 | 0 | 0.0 | 8.8 |
|  |  |  |  |  |  |  |  |  |  |  |  |  |  |  |  |  |  |  |
| Fatigue | All | 40 | 8 | 20.0 | 9.1 | 35.6 |  | 40 | 7 | 17.5 | 7.3 | 32.8 |  | 40 | 14 | 35.0 | 20.6 | 51.7 |
|  | Severe | 40 | 1 | 2.5 | 0.1 | 13.2 |  | 40 | 0 | 0 | 0.0 | 8.8 |  | 40 | 1 | 2.5 | 0.1 | 13.2 |
|  | Related | 40 | 6 | 15.0 | 5.7 | 29.8 |  | 40 | 5 | 12.5 | 4.2 | 26.8 |  | 40 | 11 | 27.5 | 14.6 | 43.9 |
|  | Severe*Related | 40 | 1 | 2.5 | 0.1 | 13.2 |  | 40 | 0 | 0 | 0.0 | 8.8 |  | 40 | 0 | 0 | 0.0 | 8.8 |
|  | Medically Attended Visit | 40 | 0 | 0 | 0.0 | 8.8 |  | 40 | 0 | 0 | 0.0 | 8.8 |  | 40 | 0 | 0 | 0.0 | 8.8 |
|  |  |  |  |  |  |  |  |  |  |  |  |  |  |  |  |  |  |  |
| Gastrointestinal Symptoms | All | 40 | 4 | 10.0 | 2.8 | 23.7 |  | 40 | 6 | 15.0 | 5.7 | 29.8 |  | 40 | 9 | 22.5 | 10.8 | 38.5 |
|  | Severe | 40 | 0 | 0 | 0.0 | 8.8 |  | 40 | 0 | 0 | 0.0 | 8.8 |  | 40 | 1 | 2.5 | 0.1 | 13.2 |
|  | Related | 40 | 2 | 5.0 | 0.6 | 16.9 |  | 40 | 6 | 15.0 | 5.7 | 29.8 |  | 40 | 6 | 15.0 | 5.7 | 29.8 |
|  | Severe*Related | 40 | 0 | 0 | 0.0 | 8.8 |  | 40 | 0 | 0 | 0.0 | 8.8 |  | 40 | 0 | 0 | 0.0 | 8.8 |
|  | Medically Attended Visit | 40 | 1 | 2.5 | 0.1 | 13.2 |  | 40 | 1 | 2.5 | 0.1 | 13.2 |  | 40 | 0 | 0 | 0.0 | 8.8 |
|  |  |  |  |  |  |  |  |  |  |  |  |  |  |  |  |  |  |  |
| Note(s): For each dose and overall/subject: N = number of subjects with at least one documented dose; n/% = number/percentage of subjects reporting at least once the symptom. For Overall/dose: N = number of documented doses; n/% = number/percentage of doses followed by at least one type of symptom. 95% CI = exact 95% confidence interval; LL = lower limit; UL = upper limit. | | | | | | | | | | | | | | | | | | |

|  | | rNV-2v [50 µg GI.4 + 50 µg GII.4] | | | | |  | rNV-2v [150 µg GI.4 + 150 µg GII.4] | | | | |  | Placebo | | | | |
| --- | --- | --- | --- | --- | --- | --- | --- | --- | --- | --- | --- | --- | --- | --- | --- | --- | --- | --- |
| Symptom | Type | N | n | % | 95%  LL | 95%  UL |  | N | n | % | 95%  LL | 95%  UL |  | N | n | % | 95%  LL | 95%  UL |
|  |  |  |  |  |  |  |  |  |  |  |  |  |  |  |  |  |  |  |
| Overall/Dose |  |  |  |  |  |  |  |  |  |  |  |  |  |  |  |  |  |  |
| Headache | All | 40 | 8 | 20.0 | 9.1 | 35.6 |  | 40 | 8 | 20.0 | 9.1 | 35.6 |  | 40 | 13 | 32.5 | 18.6 | 49.1 |
|  | Severe | 40 | 1 | 2.5 | 0.1 | 13.2 |  | 40 | 0 | 0 | 0.0 | 8.8 |  | 40 | 1 | 2.5 | 0.1 | 13.2 |
|  | Related | 40 | 5 | 12.5 | 4.2 | 26.8 |  | 40 | 6 | 15.0 | 5.7 | 29.8 |  | 40 | 9 | 22.5 | 10.8 | 38.5 |
|  | Severe*Related | 40 | 1 | 2.5 | 0.1 | 13.2 |  | 40 | 0 | 0 | 0.0 | 8.8 |  | 40 | 0 | 0 | 0.0 | 8.8 |
|  | Medically Attended Visit | 40 | 1 | 2.5 | 0.1 | 13.2 |  | 40 | 0 | 0 | 0.0 | 8.8 |  | 40 | 3 | 7.5 | 1.6 | 20.4 |
|  |  |  |  |  |  |  |  |  |  |  |  |  |  |  |  |  |  |  |
| Myalgia | All | 40 | 3 | 7.5 | 1.6 | 20.4 |  | 40 | 1 | 2.5 | 0.1 | 13.2 |  | 40 | 3 | 7.5 | 1.6 | 20.4 |
|  | Severe | 40 | 0 | 0 | 0.0 | 8.8 |  | 40 | 0 | 0 | 0.0 | 8.8 |  | 40 | 0 | 0 | 0.0 | 8.8 |
|  | Related | 40 | 1 | 2.5 | 0.1 | 13.2 |  | 40 | 0 | 0 | 0.0 | 8.8 |  | 40 | 3 | 7.5 | 1.6 | 20.4 |
|  | Severe*Related | 40 | 0 | 0 | 0.0 | 8.8 |  | 40 | 0 | 0 | 0.0 | 8.8 |  | 40 | 0 | 0 | 0.0 | 8.8 |
|  | Medically Attended Visit | 40 | 1 | 2.5 | 0.1 | 13.2 |  | 40 | 0 | 0 | 0.0 | 8.8 |  | 40 | 0 | 0 | 0.0 | 8.8 |
|  |  |  |  |  |  |  |  |  |  |  |  |  |  |  |  |  |  |  |
| Shivering | All | 40 | 2 | 5.0 | 0.6 | 16.9 |  | 40 | 0 | 0 | 0.0 | 8.8 |  | 40 | 2 | 5.0 | 0.6 | 16.9 |
|  | Severe | 40 | 0 | 0 | 0.0 | 8.8 |  | 40 | 0 | 0 | 0.0 | 8.8 |  | 40 | 0 | 0 | 0.0 | 8.8 |
|  | Related | 40 | 1 | 2.5 | 0.1 | 13.2 |  | 40 | 0 | 0 | 0.0 | 8.8 |  | 40 | 1 | 2.5 | 0.1 | 13.2 |
|  | Severe*Related | 40 | 0 | 0 | 0.0 | 8.8 |  | 40 | 0 | 0 | 0.0 | 8.8 |  | 40 | 0 | 0 | 0.0 | 8.8 |
|  | Medically Attended Visit | 40 | 0 | 0 | 0.0 | 8.8 |  | 40 | 0 | 0 | 0.0 | 8.8 |  | 40 | 0 | 0 | 0.0 | 8.8 |
|  |  |  |  |  |  |  |  |  |  |  |  |  |  |  |  |  |  |  |
| Temperature | All | 40 | 0 | 0 | 0.0 | 8.8 |  | 40 | 0 | 0 | 0.0 | 8.8 |  | 40 | 0 | 0 | 0.0 | 8.8 |
|  |  |  |  |  |  |  |  |  |  |  |  |  |  |  |  |  |  |  |
| Note(s): For each dose and overall/subject: N = number of subjects with at least one documented dose; n/% = number/percentage of subjects reporting at least once the symptom. For Overall/dose: N = number of documented doses; n/% = number/percentage of doses followed by at least one type of symptom. 95% CI = exact 95% confidence interval; LL = lower limit; UL = upper limit. | | | | | | | | | | | | | | | | | | |

|  | | rNV-2v [50 µg GI.4 + 50 µg GII.4] | | | | |  | rNV-2v [150 µg GI.4 + 150 µg GII.4] | | | | |  | Placebo | | | | |
| --- | --- | --- | --- | --- | --- | --- | --- | --- | --- | --- | --- | --- | --- | --- | --- | --- | --- | --- |
| Symptom | Type | N | n | % | 95%  LL | 95%  UL |  | N | n | % | 95%  LL | 95%  UL |  | N | n | % | 95%  LL | 95%  UL |
|  |  |  |  |  |  |  |  |  |  |  |  |  |  |  |  |  |  |  |
| Overall/Subject |  |  |  |  |  |  |  |  |  |  |  |  |  |  |  |  |  |  |
| Arthralgia | All | 20 | 0 | 0 | 0.0 | 16.8 |  | 20 | 1 | 5.0 | 0.1 | 24.9 |  | 20 | 0 | 0 | 0.0 | 16.8 |
|  | Severe | 20 | 0 | 0 | 0.0 | 16.8 |  | 20 | 0 | 0 | 0.0 | 16.8 |  | 20 | 0 | 0 | 0.0 | 16.8 |
|  | Related | 20 | 0 | 0 | 0.0 | 16.8 |  | 20 | 0 | 0 | 0.0 | 16.8 |  | 20 | 0 | 0 | 0.0 | 16.8 |
|  | Severe*Related | 20 | 0 | 0 | 0.0 | 16.8 |  | 20 | 0 | 0 | 0.0 | 16.8 |  | 20 | 0 | 0 | 0.0 | 16.8 |
|  | Medically Attended Visit | 20 | 0 | 0 | 0.0 | 16.8 |  | 20 | 0 | 0 | 0.0 | 16.8 |  | 20 | 0 | 0 | 0.0 | 16.8 |
|  |  |  |  |  |  |  |  |  |  |  |  |  |  |  |  |  |  |  |
| Fatigue | All | 20 | 6 | 30.0 | 11.9 | 54.3 |  | 20 | 5 | 25.0 | 8.7 | 49.1 |  | 20 | 9 | 45.0 | 23.1 | 68.5 |
|  | Severe | 20 | 1 | 5.0 | 0.1 | 24.9 |  | 20 | 0 | 0 | 0.0 | 16.8 |  | 20 | 1 | 5.0 | 0.1 | 24.9 |
|  | Related | 20 | 5 | 25.0 | 8.7 | 49.1 |  | 20 | 4 | 20.0 | 5.7 | 43.7 |  | 20 | 8 | 40.0 | 19.1 | 63.9 |
|  | Severe*Related | 20 | 1 | 5.0 | 0.1 | 24.9 |  | 20 | 0 | 0 | 0.0 | 16.8 |  | 20 | 0 | 0 | 0.0 | 16.8 |
|  | Medically Attended Visit | 20 | 0 | 0 | 0.0 | 16.8 |  | 20 | 0 | 0 | 0.0 | 16.8 |  | 20 | 0 | 0 | 0.0 | 16.8 |
|  |  |  |  |  |  |  |  |  |  |  |  |  |  |  |  |  |  |  |
| Gastrointestinal Symptoms | All | 20 | 4 | 20.0 | 5.7 | 43.7 |  | 20 | 4 | 20.0 | 5.7 | 43.7 |  | 20 | 7 | 35.0 | 15.4 | 59.2 |
|  | Severe | 20 | 0 | 0 | 0.0 | 16.8 |  | 20 | 0 | 0 | 0.0 | 16.8 |  | 20 | 1 | 5.0 | 0.1 | 24.9 |
|  | Related | 20 | 2 | 10.0 | 1.2 | 31.7 |  | 20 | 4 | 20.0 | 5.7 | 43.7 |  | 20 | 5 | 25.0 | 8.7 | 49.1 |
|  | Severe*Related | 20 | 0 | 0 | 0.0 | 16.8 |  | 20 | 0 | 0 | 0.0 | 16.8 |  | 20 | 0 | 0 | 0.0 | 16.8 |
|  | Medically Attended Visit | 20 | 1 | 5.0 | 0.1 | 24.9 |  | 20 | 1 | 5.0 | 0.1 | 24.9 |  | 20 | 0 | 0 | 0.0 | 16.8 |
|  |  |  |  |  |  |  |  |  |  |  |  |  |  |  |  |  |  |  |
| Note(s): For each dose and overall/subject: N = number of subjects with at least one documented dose; n/% = number/percentage of subjects reporting at least once the symptom. For Overall/dose: N = number of documented doses; n/% = number/percentage of doses followed by at least one type of symptom. 95% CI = exact 95% confidence interval; LL = lower limit; UL = upper limit. | | | | | | | | | | | | | | | | | | |

|  | | rNV-2v [50 µg GI.4 + 50 µg GII.4] | | | | |  | rNV-2v [150 µg GI.4 + 150 µg GII.4] | | | | |  | Placebo | | | | |
| --- | --- | --- | --- | --- | --- | --- | --- | --- | --- | --- | --- | --- | --- | --- | --- | --- | --- | --- |
| Symptom | Type | N | n | % | 95%  LL | 95%  UL |  | N | n | % | 95%  LL | 95%  UL |  | N | n | % | 95%  LL | 95%  UL |
|  |  |  |  |  |  |  |  |  |  |  |  |  |  |  |  |  |  |  |
| Overall/Subject |  |  |  |  |  |  |  |  |  |  |  |  |  |  |  |  |  |  |
| Headache | All | 20 | 7 | 35.0 | 15.4 | 59.2 |  | 20 | 7 | 35.0 | 15.4 | 59.2 |  | 20 | 10 | 50.0 | 27.2 | 72.8 |
|  | Severe | 20 | 1 | 5.0 | 0.1 | 24.9 |  | 20 | 0 | 0 | 0.0 | 16.8 |  | 20 | 1 | 5.0 | 0.1 | 24.9 |
|  | Related | 20 | 5 | 25.0 | 8.7 | 49.1 |  | 20 | 5 | 25.0 | 8.7 | 49.1 |  | 20 | 8 | 40.0 | 19.1 | 63.9 |
|  | Severe*Related | 20 | 1 | 5.0 | 0.1 | 24.9 |  | 20 | 0 | 0 | 0.0 | 16.8 |  | 20 | 0 | 0 | 0.0 | 16.8 |
|  | Medically Attended Visit | 20 | 1 | 5.0 | 0.1 | 24.9 |  | 20 | 0 | 0 | 0.0 | 16.8 |  | 20 | 3 | 15.0 | 3.2 | 37.9 |
|  |  |  |  |  |  |  |  |  |  |  |  |  |  |  |  |  |  |  |
| Myalgia | All | 20 | 3 | 15.0 | 3.2 | 37.9 |  | 20 | 1 | 5.0 | 0.1 | 24.9 |  | 20 | 3 | 15.0 | 3.2 | 37.9 |
|  | Severe | 20 | 0 | 0 | 0.0 | 16.8 |  | 20 | 0 | 0 | 0.0 | 16.8 |  | 20 | 0 | 0 | 0.0 | 16.8 |
|  | Related | 20 | 1 | 5.0 | 0.1 | 24.9 |  | 20 | 0 | 0 | 0.0 | 16.8 |  | 20 | 3 | 15.0 | 3.2 | 37.9 |
|  | Severe*Related | 20 | 0 | 0 | 0.0 | 16.8 |  | 20 | 0 | 0 | 0.0 | 16.8 |  | 20 | 0 | 0 | 0.0 | 16.8 |
|  | Medically Attended Visit | 20 | 1 | 5.0 | 0.1 | 24.9 |  | 20 | 0 | 0 | 0.0 | 16.8 |  | 20 | 0 | 0 | 0.0 | 16.8 |
|  |  |  |  |  |  |  |  |  |  |  |  |  |  |  |  |  |  |  |
| Shivering | All | 20 | 2 | 10.0 | 1.2 | 31.7 |  | 20 | 0 | 0 | 0.0 | 16.8 |  | 20 | 2 | 10.0 | 1.2 | 31.7 |
|  | Severe | 20 | 0 | 0 | 0.0 | 16.8 |  | 20 | 0 | 0 | 0.0 | 16.8 |  | 20 | 0 | 0 | 0.0 | 16.8 |
|  | Related | 20 | 1 | 5.0 | 0.1 | 24.9 |  | 20 | 0 | 0 | 0.0 | 16.8 |  | 20 | 1 | 5.0 | 0.1 | 24.9 |
|  | Severe*Related | 20 | 0 | 0 | 0.0 | 16.8 |  | 20 | 0 | 0 | 0.0 | 16.8 |  | 20 | 0 | 0 | 0.0 | 16.8 |
|  | Medically Attended Visit | 20 | 0 | 0 | 0.0 | 16.8 |  | 20 | 0 | 0 | 0.0 | 16.8 |  | 20 | 0 | 0 | 0.0 | 16.8 |
|  |  |  |  |  |  |  |  |  |  |  |  |  |  |  |  |  |  |  |
| Temperature | All | 20 | 0 | 0 | 0.0 | 16.8 |  | 20 | 0 | 0 | 0.0 | 16.8 |  | 20 | 0 | 0 | 0.0 | 16.8 |
|  |  |  |  |  |  |  |  |  |  |  |  |  |  |  |  |  |  |  |
| Note(s): For each dose and overall/subject: N = number of subjects with at least one documented dose; n/% = number/percentage of subjects reporting at least once the symptom. For Overall/dose: N = number of documented doses; n/% = number/percentage of doses followed by at least one type of symptom. 95% CI = exact 95% confidence interval; LL = lower limit; UL = upper limit. | | | | | | | | | | | | | | | | | | |

| **Supplementary Table 5:** Number and Percentage of Subjects Reporting the Occurrence of Unsolicited Adverse Events Classified by MedDRA Primary System Organ Class and Preferred Term During the 28-day Post-vaccination Period | | | | | | | | | | | | | | | | | |
| --- | --- | --- | --- | --- | --- | --- | --- | --- | --- | --- | --- | --- | --- | --- | --- | --- | --- |
|  | rNV-2v [50 µg GI.4 + 50 µg GII.4] | | | | |  | rNV-2v [150 µg GI.4 + 150 µg GII.4] | | | | |  | Placebo | | | | |
| SYSTEM ORGAN CLASS/   Preferred Term | N | n | % | 95% LL | 95% UL |  | N | n | % | 95% LL | 95% UL |  | N | n | % | 95% LL | 95% UL |
|  |  |  |  |  |  |  |  |  |  |  |  |  |  |  |  |  |  |
| At least one unsolicited adverse events | 20 | 8 | 40.0 | 19.1 | 63.9 |  | 20 | 8 | 40.0 | 19.1 | 63.9 |  | 20 | 8 | 40.0 | 19.1 | 63.9 |
|  |  |  |  |  |  |  |  |  |  |  |  |  |  |  |  |  |  |
| GASTROINTESTINAL DISORDERS | 20 | 1 | 5.0 | 0.1 | 24.9 |  | 20 | 3 | 15.0 | 3.2 | 37.9 |  | 20 | 0 | 0 | 0.0 | 16.8 |
| Dyspepsia | 20 | 0 | 0 | 0.0 | 16.8 |  | 20 | 1 | 5.0 | 0.1 | 24.9 |  | 20 | 0 | 0 | 0.0 | 16.8 |
| Gastritis | 20 | 0 | 0 | 0.0 | 16.8 |  | 20 | 1 | 5.0 | 0.1 | 24.9 |  | 20 | 0 | 0 | 0.0 | 16.8 |
| Gastrooesophageal reflux disease | 20 | 1 | 5.0 | 0.1 | 24.9 |  | 20 | 0 | 0 | 0.0 | 16.8 |  | 20 | 0 | 0 | 0.0 | 16.8 |
| Mouth ulceration | 20 | 0 | 0 | 0.0 | 16.8 |  | 20 | 1 | 5.0 | 0.1 | 24.9 |  | 20 | 0 | 0 | 0.0 | 16.8 |
|  |  |  |  |  |  |  |  |  |  |  |  |  |  |  |  |  |  |
| GENERAL DISORDERS AND ADMINISTRATION SITE CONDITIONS | 20 | 1 | 5.0 | 0.1 | 24.9 |  | 20 | 2 | 10.0 | 1.2 | 31.7 |  | 20 | 0 | 0 | 0.0 | 16.8 |
| Fatigue | 20 | 0 | 0 | 0.0 | 16.8 |  | 20 | 1 | 5.0 | 0.1 | 24.9 |  | 20 | 0 | 0 | 0.0 | 16.8 |
| Injection site pruritus | 20 | 0 | 0 | 0.0 | 16.8 |  | 20 | 1 | 5.0 | 0.1 | 24.9 |  | 20 | 0 | 0 | 0.0 | 16.8 |
| Non-cardiac chest pain | 20 | 1 | 5.0 | 0.1 | 24.9 |  | 20 | 0 | 0 | 0.0 | 16.8 |  | 20 | 0 | 0 | 0.0 | 16.8 |
|  |  |  |  |  |  |  |  |  |  |  |  |  |  |  |  |  |  |
| INFECTIONS AND INFESTATIONS | 20 | 2 | 10.0 | 1.2 | 31.7 |  | 20 | 3 | 15.0 | 3.2 | 37.9 |  | 20 | 3 | 15.0 | 3.2 | 37.9 |
| Gastroenteritis | 20 | 0 | 0 | 0.0 | 16.8 |  | 20 | 1 | 5.0 | 0.1 | 24.9 |  | 20 | 1 | 5.0 | 0.1 | 24.9 |
| Nasopharyngitis | 20 | 0 | 0 | 0.0 | 16.8 |  | 20 | 0 | 0 | 0.0 | 16.8 |  | 20 | 1 | 5.0 | 0.1 | 24.9 |
| Pharyngotonsillitis | 20 | 0 | 0 | 0.0 | 16.8 |  | 20 | 1 | 5.0 | 0.1 | 24.9 |  | 20 | 0 | 0 | 0.0 | 16.8 |
| Tonsillitis | 20 | 0 | 0 | 0.0 | 16.8 |  | 20 | 1 | 5.0 | 0.1 | 24.9 |  | 20 | 1 | 5.0 | 0.1 | 24.9 |
| Upper respiratory tract infection | 20 | 1 | 5.0 | 0.1 | 24.9 |  | 20 | 0 | 0 | 0.0 | 16.8 |  | 20 | 0 | 0 | 0.0 | 16.8 |
| Vulval abscess | 20 | 1 | 5.0 | 0.1 | 24.9 |  | 20 | 0 | 0 | 0.0 | 16.8 |  | 20 | 0 | 0 | 0.0 | 16.8 |
|  |  |  |  |  |  |  |  |  |  |  |  |  |  |  |  |  |  |
| MUSCULOSKELETAL AND CONNECTIVE TISSUE DISORDERS | 20 | 3 | 15.0 | 3.2 | 37.9 |  | 20 | 1 | 5.0 | 0.1 | 24.9 |  | 20 | 0 | 0 | 0.0 | 16.8 |
| Back pain | 20 | 1 | 5.0 | 0.1 | 24.9 |  | 20 | 1 | 5.0 | 0.1 | 24.9 |  | 20 | 0 | 0 | 0.0 | 16.8 |
| Muscle spasms | 20 | 1 | 5.0 | 0.1 | 24.9 |  | 20 | 0 | 0 | 0.0 | 16.8 |  | 20 | 0 | 0 | 0.0 | 16.8 |
| Tendonitis | 20 | 1 | 5.0 | 0.1 | 24.9 |  | 20 | 0 | 0 | 0.0 | 16.8 |  | 20 | 0 | 0 | 0.0 | 16.8 |
|  |  |  |  |  |  |  |  |  |  |  |  |  |  |  |  |  |  |
| Note(s): Adverse events were coded using the MedDRA coding dictionary, version 24.1. At least one symptom = at least one symptom experienced (regardless of the MedDRA Preferred Term); MedDRA = medical dictionary for regulatory activities. N = number of subjects with at least one administered dose. n/% = number/percentage of subjects reporting the unsolicited adverse events at least once. 95% CI = exact 95% confidence interval; LL = lower limit; UL = upper limit. | | | | | | | | | | | | | | | | | |

|  | rNV-2v [50 µg GI.4 + 50 µg GII.4] | | | | |  | rNV-2v [150 µg GI.4 + 150 µg GII.4] | | | | |  | Placebo | | | | |
| --- | --- | --- | --- | --- | --- | --- | --- | --- | --- | --- | --- | --- | --- | --- | --- | --- | --- |
| SYSTEM ORGAN CLASS/   Preferred Term | N | n | % | 95% LL | 95% UL |  | N | n | % | 95% LL | 95% UL |  | N | n | % | 95% LL | 95% UL |
|  |  |  |  |  |  |  |  |  |  |  |  |  |  |  |  |  |  |
| NERVOUS SYSTEM DISORDERS | 20 | 2 | 10.0 | 1.2 | 31.7 |  | 20 | 1 | 5.0 | 0.1 | 24.9 |  | 20 | 6 | 30.0 | 11.9 | 54.3 |
| Dizziness | 20 | 0 | 0 | 0.0 | 16.8 |  | 20 | 0 | 0 | 0.0 | 16.8 |  | 20 | 3 | 15.0 | 3.2 | 37.9 |
| Headache | 20 | 2 | 10.0 | 1.2 | 31.7 |  | 20 | 1 | 5.0 | 0.1 | 24.9 |  | 20 | 4 | 20.0 | 5.7 | 43.7 |
|  |  |  |  |  |  |  |  |  |  |  |  |  |  |  |  |  |  |
| RESPIRATORY, THORACIC AND MEDIASTINAL DISORDERS | 20 | 1 | 5.0 | 0.1 | 24.9 |  | 20 | 1 | 5.0 | 0.1 | 24.9 |  | 20 | 3 | 15.0 | 3.2 | 37.9 |
| Cough | 20 | 0 | 0 | 0.0 | 16.8 |  | 20 | 0 | 0 | 0.0 | 16.8 |  | 20 | 1 | 5.0 | 0.1 | 24.9 |
| Epistaxis | 20 | 0 | 0 | 0.0 | 16.8 |  | 20 | 0 | 0 | 0.0 | 16.8 |  | 20 | 1 | 5.0 | 0.1 | 24.9 |
| Oropharyngeal pain | 20 | 1 | 5.0 | 0.1 | 24.9 |  | 20 | 0 | 0 | 0.0 | 16.8 |  | 20 | 1 | 5.0 | 0.1 | 24.9 |
| Rhinorrhoea | 20 | 0 | 0 | 0.0 | 16.8 |  | 20 | 1 | 5.0 | 0.1 | 24.9 |  | 20 | 0 | 0 | 0.0 | 16.8 |
|  |  |  |  |  |  |  |  |  |  |  |  |  |  |  |  |  |  |
| Note(s): Adverse events were coded using the MedDRA coding dictionary, version 24.1. At least one symptom = at least one symptom experienced (regardless of the MedDRA Preferred Term); MedDRA = medical dictionary for regulatory activities. N = number of subjects with at least one administered dose. n/% = number/percentage of subjects reporting the unsolicited adverse events at least once. 95% CI = exact 95% confidence interval; LL = lower limit; UL = upper limit. | | | | | | | | | | | | | | | | | |

Supplementary Table 6: Stability data at +5 ± 3°C for rNV-2v clinical batches

| **Test** | | **Acceptance Criterion** | **Method** | **Time Points** | | | | |
| --- | --- | --- | --- | --- | --- | --- | --- | --- |
|  |  |  |  | **Initial** | **3 months** | **6 months** | **9 months** | **12 months** |
| **rNV-2v**  **50 µg GI.4 + 50 µg GII.4** | Total protein content (mg/mL) | 0.2 ± 0.1 mg/mL | Ph. Eur 2.5.33  (BCA with TCA  precipitation) | 0.24 | 0.22 | 0.25 | 0.19 | 0.20 |
|  | VLP content (%) | ≥ 85% | SE-HPLC, UV  detection | 97.3 | 94.5 | 93.1 | 93.0 | 93.3 |
|  | VP1 purity (%) | ≥ 90% | Capillary Gel Electrophoresis, reduced | 100 | 100 | 100 | 100 | 100 |
| **rNV-2v**  **150 µg GI.4 + 150 µg GII.4** | Total protein content (mg/mL) | 0.6 ± 0.1 mg/mL | Ph. Eur 2.5.33  (BCA with TCA  precipitation) | 0.59 | 0.58 | 0.59 | 0.50 | 0.52 |
|  | VLP content (%) | ≥ 85% | SE-HPLC, UV  detection | 97.7 | 96.8 | 97.5 | 97.1 | 97.0 |
|  | VP1 purity (%) | ≥ 90% | Capillary Gel Electrophoresis, reduced | 100 | 99.2 | 99.3 | 100 | 98.5 |

Supplementary Figure 1: Schematic of Study Design

**Cohort 2^a, b^**

**150 µg VLP GI.4/150 µg VLP GII.4/Placebo
Intramuscular 0.5 mL injection**

**Dose 1 (Day 1)**

**Cohort 2^a, b^**

**150 µg VLP GI.4/150 µg VLP GII.4/Placebo
Intramuscular 0.5 mL injection**

**Dose 2 (Day 29)**

**Cohort 1^a, b^**

**50 µg VLP GI.4/50 µg VLP GII.4/Placebo
Intramuscular 0.5 mL injection**

**Dose 1 (Day 1)**

**Cohort 1^a, b^**

**50 µg VLP GI.4/50 µg VLP GII.4/Placebo
Intramuscular 0.5 mL injection**

**Dose 2 (Day 29)**

The SRC reviewed all available safety data from the first 7 days of at least 20 of the 30 subjects in Cohort 1 and Cohort 2 post-first dose of the IMP, before proceeding to the second dose. The SRC reviewed available safety data from the 30 subjects in Cohort 1 (all data up to 7 days post second dose of the IMP), before proceeding to Cohort 2.

AE = adverse event, IMP = investigational medicinal product, PI = Principal Investigator, SRC = Safety Review Committee, VLP = virus-like particle

Note: The 2 doses of the IMP in each cohort were administered 1 month apart.

a The first 3 subjects (2 active and 1 placebo) in each cohort were dosed on Day 1. Following a review of the safety data obtained after at least 48 hours by the PI, and in the absence of any safety concerns, 3 additional subjects (2 active and 1 placebo) were to be dosed. The safety data of all sentinel subjects were obtained through a safety phone call at least 48 hours after dosing. All safety data of the first 6 subjects were reviewed by the PI. If no safety or tolerability concerns arose during this review, the remainder of the subjects in the cohort (24 subjects) were to be dosed. A maximum of 12 subjects were dosed on a single day. The same sentinel strategy was followed for the second dose of the IMP in both cohorts.

b A safety visit was conducted 7 days after each administration of IMP to perform laboratory assessments, safety tests, and to review AEs (including subject diary cards). Serious adverse events and onset of any significant new medical conditions were followed until the event was resolved, stabilized, otherwise explained, or the subject was lost to follow-up.

Supplementary Figure 2: Sentinel Dosing – rNV-2v (Cohort 1 and Cohort 2)

Day 9

Dosing of Remaining Subjects (12 subjects)

**Day 1**

First Sentinel Dosing
(2 active/1 placebo)

**Day 5**

Second Sentinel Dosing

(2 Active/1 Placebo)

**Days 2 and 3**

Observation Period

**Days 6 and 7**

Observation Period

**Day 4**

Safety Evaluation by the PI

**Day 8**

Safety Evaluation by the PI

**Day 10**

Dosing of Remaining Subjects (12 subjects)

PI = Principal Investigator

Note:

This dosing schedule is a simulation considering the Day 1 visit on a Monday. Changes to the schedule could be made to avoid any study visit on a Saturday/Sunday (weekend).

The sentinel dosing strategy was applied for both first and second doses of the investigational medicinal product (IMP) in each cohort.

Supplementary Figure 3: Evolutionary Relationships of Virus Protein 1 Protruding Domains amino acid sequence of Norovirus Virus-like Particles used in Enzyme-linked Immunosorbent Assays to Assess Cross-reactive Immunoglobulin G


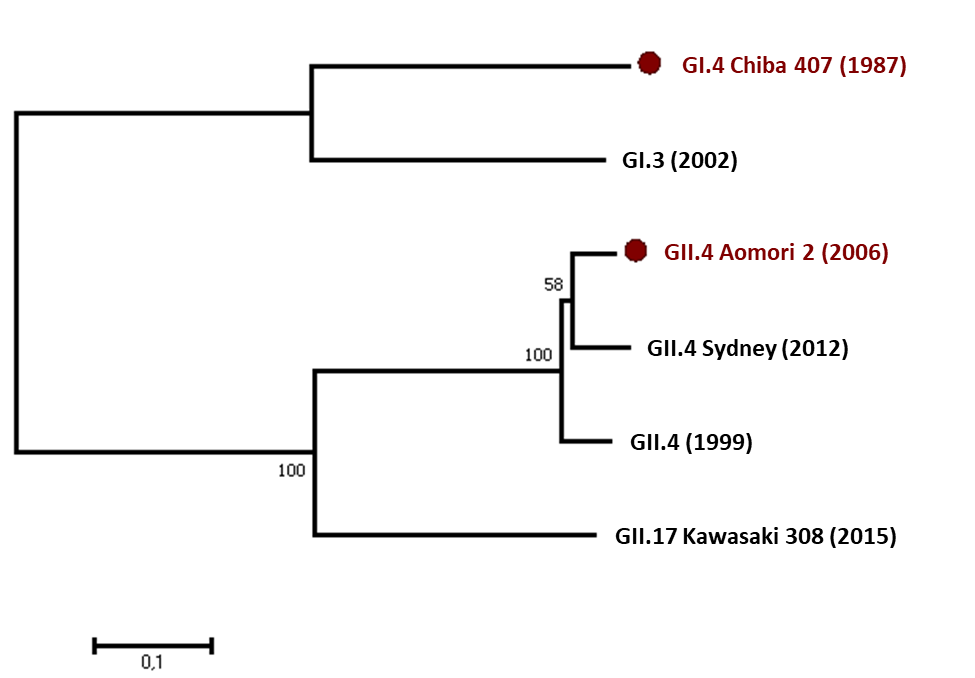


The evolutionary history was inferred using the Neighbor-Joining method [1] on an alignment generated with MUSCLE [2]. The optimal tree with the sum of branch length = 1,60049444 is shown. The percentage of replicate trees in which the associated taxa clustered together in the bootstrap test (10000 replicates) are shown next to the branches [3]. The tree is drawn to scale, with branch lengths in the same units as those of the evolutionary distances used to infer the phylogenetic tree. The evolutionary distances were computed using the Poisson correction method [4] and are in the units of the number of amino acid substitutions per site. The analysis involved 6 amino acid sequences. A total of 300 positions is considered in the final dataset. Evolutionary analyses were conducted in MEGA7 [5].

1. Saitou N. and Nei M. (1987). The neighbor-joining method: A new method for reconstructing phylogenetic trees. Molecular Biology and Evolution 4:406-425.
2. Edgar, Robert C. (2004), MUSCLE: multiple sequence alignment with high accuracy and high throughput, Nucleic Acids Research 32(5), 1792-97
3. Felsenstein J. (1985). Confidence limits on phylogenies: An approach using the bootstrap. Evolution 39:783-791.
4. Zuckerkandl E. and Pauling L. (1965). Evolutionary divergence and convergence in proteins. Edited in Evolving Genes and Proteins by V. Bryson and H.J. Vogel, pp. 97-166. Academic Press, New York.
5. Kumar S., Stecher G., and Tamura K. (2016). MEGA7: Molecular Evolutionary Genetics Analysis version 7.0 for bigger datasets. Molecular Biology and Evolution 33:1870-1874.
